# Tumor cell–derived spermidine promotes a protumorigenic immune microenvironment in glioblastoma via CD8^+^ T cell inhibition

**DOI:** 10.1172/JCI177824

**Published:** 2024-11-19

**Authors:** Kristen E. Kay, Juyeun Lee, Ellen S. Hong, Julia Beilis, Sahil Dayal, Emily R. Wesley, Sofia Mitchell, Sabrina Z. Wang, Daniel J. Silver, Josephine Volovetz, Sadie Johnson, Mary McGraw, Matthew M. Grabowski, Tianyao Lu, Lutz Freytag, Vinod Narayana, Saskia Freytag, Sarah A. Best, James R. Whittle, Zeneng Wang, Ofer Reizes, Jennifer S. Yu, Stanley L. Hazen, J. Mark Brown, Defne Bayik, Justin D. Lathia

**Affiliations:** 1 Department of Cardiovascular and Metabolic Sciences, Lerner Research Institute, Cleveland Clinic, Cleveland, Ohio, USA.; 2 Cleveland Clinic Lerner College of Medicine; and; 3 Medical Scientist Training Program, School of Medicine; Case Western Reserve University, Cleveland, Ohio, USA.; 4 Rose Ella Burkhardt Brain Tumor Center, Cleveland Clinic, Cleveland, Ohio, USA.; 5 Case Comprehensive Cancer Center, Cleveland, Ohio, USA.; 6 Personalised Oncology Division, The Walter and Eliza Hall Institute of Medical Research, Melbourne, Victoria, Australia.; 7 Department of Medical Biology; and; 8 Metabolomics Australia, Bio21 Molecular Science and Biotechnology Institute; The University of Melbourne, Melbourne, Victoria, Australia.; 9 Department of Medical Oncology, Peter MacCallum Cancer Centre, Melbourne, Victoria, Australia.; 10 Department of Cancer Biology, Lerner Research Institute, Cleveland Clinic, Cleveland, Ohio, USA.; 11 Department of Molecular and Cellular Pharmacology, Miller School of Medicine; and; 12 Sylvester Comprehensive Cancer Center; University of Miami, Miami, Florida, USA.

**Keywords:** Immunology, Oncology, Adaptive immunity, Brain cancer, Polyamines

## Abstract

The glioblastoma (GBM) microenvironment is enriched in immunosuppressive factors that potently interfere with the function of cytotoxic T lymphocytes. Cancer cells can directly affect the immune system, but the mechanisms driving these interactions are not completely clear. Here, we demonstrate that the polyamine metabolite spermidine (SPD) was elevated in the GBM tumor microenvironment. Exogenous administration of SPD drove tumor aggressiveness in an immune-dependent manner in preclinical mouse models via reduction of CD8^+^ T cell frequency and reduced cytotoxic function. Knockdown of ornithine decarboxylase, the rate-limiting enzyme in SPD synthesis, did not affect cancer cell growth in vitro but did result in extended survival. Furthermore, patients with GBM with a more favorable outcome had a significant reduction in SPD compared with patients with a poor prognosis. Our results demonstrate that SPD functions as a cancer cell–derived metabolite that drives tumor progression by reducing CD8^+^ T cell numbers and function.

## Abstract 2

Tumor cell produced metabolite spermidine affects adaptive immune cells present in the tumor microenvironment, leading to enhanced immunosuppression.

## Graphical abstract

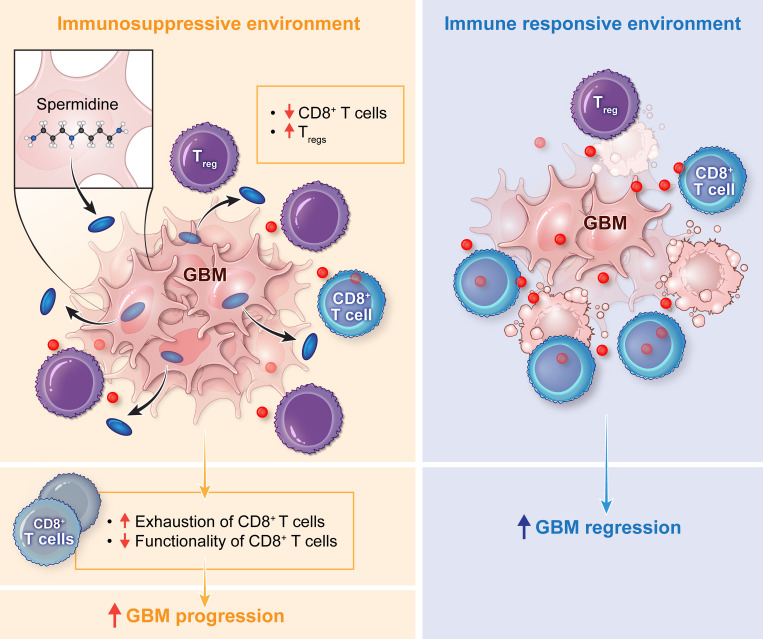

## Introduction

Despite aggressive multimodal therapies including maximal safe surgical resection followed by concomitant radiation and chemotherapy, patients with glioblastoma (GBM), the most common primary malignant brain tumor, continue to have a poor prognosis (1–3). While advances, including targeted therapies and, more recently, immunotherapy, have been achieved in other advanced cancers, GBM outcomes have not changed dramatically in decades (4–6). GBM remains a major clinical challenge owing to a variety of unique barriers, including inherent tumor cell therapeutic resistance, an immunosuppressive microenvironment, and metabolic adaptability (7–10). In particular, the tumor microenvironment contains elevated numbers of immunosuppressive cells and a limited number of effector cells (11,12). Moreover, tumor cells leverage bidirectional communication mechanisms to alter the immune microenvironment (13,14). A better understanding of these communication mechanisms in the context of immune cell infiltration, as well as their impact on the balance between immune activation and suppression, is critical for a better understanding not only of the immune microenvironment but also of the tumor’s response within.

Metabolic alterations are a hallmark of cancer and are well characterized in GBM cells. Such changes include specific dependencies involving glycolysis and lipid metabolism (15,16). Recent studies have demonstrated that metabolic programs are not static but are subject to plasticity and underlie cellular states that drive tumor growth and therapeutic resistance (17). Metabolic alterations extend beyond tumor cells and impact immune cells as well, altering their function (18). These immune cell–specific metabolic changes are triggered by the tumor microenvironment as well as tumor cells, representing another important cell communication mechanism that can alter tumor growth (19).

Polyamines are a family of cationic metabolites that include putrescine, spermine, and spermidine. These metabolites can be generated from arginine and are produced by nearly every cell in the body. Polyamines are critical to many cellular homeostatic functions, including cell growth and proliferation through their role in DNA replication and translation (20). In many cancers, including GBM, polyamines are elevated and support cancer cell growth and immune suppression (21). Specifically, in GBM, it has been shown that the polyamine family member spermidine (SPD) increases the acidity of the tumor microenvironment, shifting the balance toward immunosuppressive myeloid cells (22). Targeting the polyamine pathway at the rate-limiting step in biosynthesis has been demonstrated to increase survival in preclinical models of neuroblastoma and to synergize with conventional immune checkpoint inhibitor–based immunotherapies (23). In pediatric gliomas, additional preclinical benefit was observed using a polyamine transport inhibitor in conjunction with biosynthesis disruption (24). While these and other studies have demonstrated elevation of polyamines in GBM and a function in brain tumors, mainly involving myeloid cells, the specific sources of polyamines and the impact on the immune system as a whole are less clear. Here we show that increased SPD in the tumor microenvironment, produced in part from cancer cells, drives tumor progression by decreasing CD8^+^ T cell frequency and activity via decreased cytokine production and increased apoptosis-induced death of CD8^+^ T cells.

## Results

### SPD drives GBM progression.

**Figure 1:**
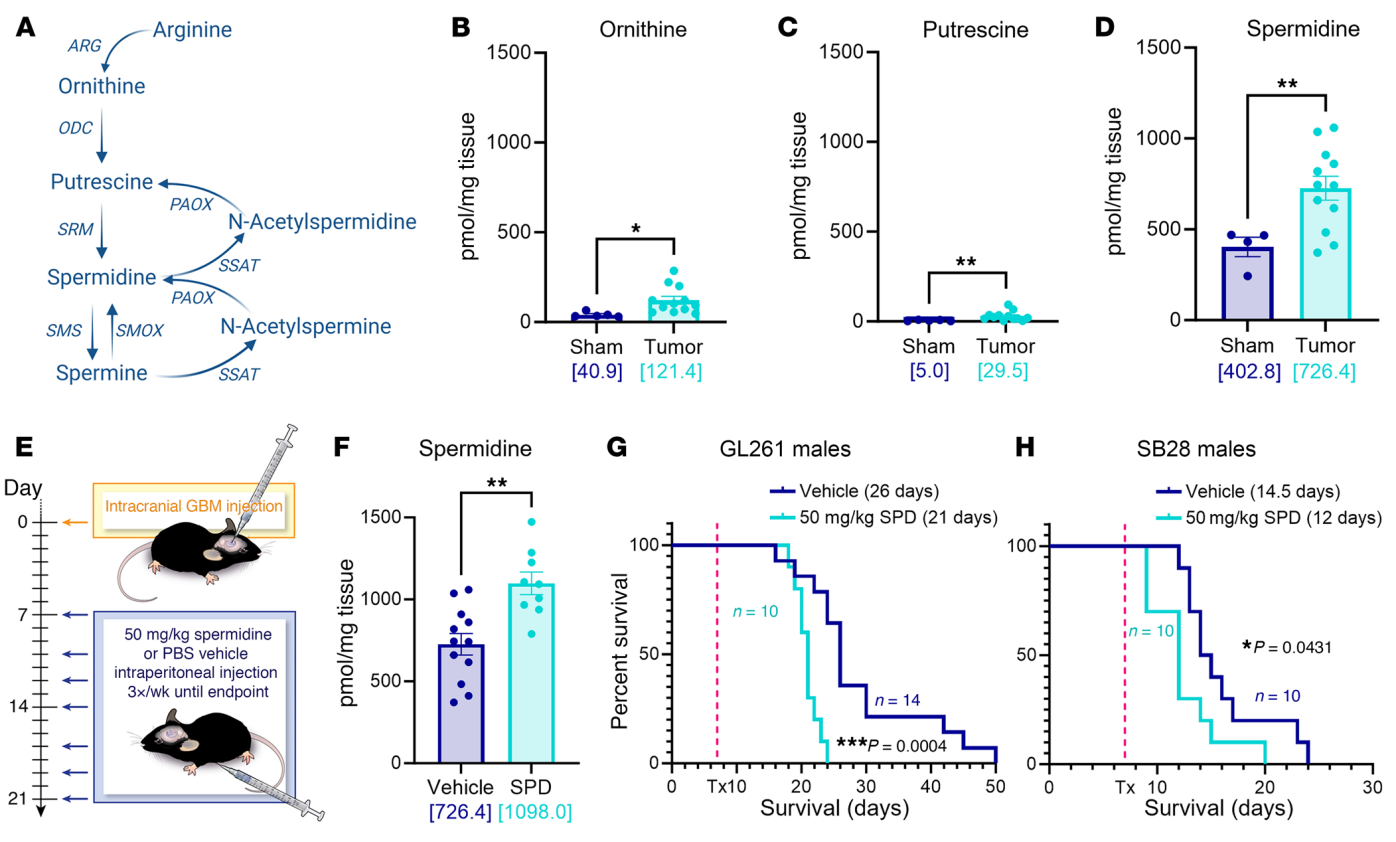
SPD levels are increased in mouse GBM models and drive GBM progression. (**A**) Polyamine biosynthesis pathway. ARG, arginase; ODC, ornithine decarboxylase; PAOX, polyamine oxidase; SMOX, spermidine oxidase; SRM, spermidine synthase; SMS, spermine synthase; SSAT, spermidine/spermine acetyltransferase. (**B**–**D**) Liquid chromatography–mass spectrometry was performed on tumors removed from male B6 mice 17 days after intracranial injection of mouse GBM cell lines (25,000 GL261 cells per injection). (**E**) Experimental paradigm for subsequent mouse experiments receiving tumor implantation followed by 50 mg/kg SPD i.p. treatment or PBS vehicle. (**F**) Liquid chromatography–tandem mass spectrometry of tumor-bearing hemisphere of mice treated with i.p. SPD. (**G** and **H**) Survival analysis was performed after intracranial injection of mouse GBM cell lines (25,000 GL261 cells per injection, 20,000 SB28 cells per injection) in B6 mice. Median survival days and number of animals are indicated in the graphs. Data combined from 3 independent experiments. Statistical significance for **B**–**D** and **F** was determined by unpaired, 2-tailed *t* test (*P < 0.05, **P < 0.01). Statistical significance for **G** and **H** was determined by log-rank test, considering *P* values less than 0.05 to be significant. Bracketed numbers indicate the mean.

It has previously been reported that patients with GBM have increased SPD in their cerebrospinal fluid and blood compared with healthy controls (25). To investigate the extent to which this is paralleled in our preclinical mouse models, we intracranially implanted the mouse glioma models SB28 and GL261 into wild-type C57BL/6 mice. Mass spectrometry of tumor tissue from these mice revealed an increase in members of the polyamine family, including a substantial increase in SPD in the tumor setting compared with control conditions. We also observed a higher-magnitude elevation in SPD in the brain compared with other polyamine family members (Figure 1, A–D, and Supplemental Figure 3, A–I; supplemental material available online with this article; https://doi.org/10.1172/JCI177824DS1). Furthermore, spatial MALDI-TOF analysis of an independent GL261 glioma model revealed tumor-intrinsic production of SPD (Supplemental Figure 1, A–E) and related enzymes in a second mouse model, CT-2A (Supplemental Figure 2, A–G). Increased SPD levels in our tumor samples compared with sham via mass spectrometry indicated that there was glioma-specific accumulation of SPD within the brain, further supported by spatial MALDI-TOF analysis. Mass spectrometry of conditioned medium of the syngeneic mouse tumor cells showed that they secreted SPD into the tumor microenvironment (Supplemental Figure 1F). These findings corroborate previous observations in human patients and suggest that SPD is increased in the tumor microenvironment. Based on the sex differences observed in GBM, both epidemiologically and in terms of immune responses (26, 27), we assessed equal numbers of male and female mice and did not observe any substantial sex differences (Figure 1, B–D, and Supplemental Figure 3, A–E). Given the lack of sex differences in response to SPD and the higher incidence and poorer outcome of GBM in males (28), we focused on males for the subsequent studies. In order to explore what effect elevated SPD would have on tumor growth, we developed an experimental paradigm in which we intracranially implanted mouse glioma cells, as previously described, and administered SPD at regular intervals via intraperitoneal injection (Figure 1E). We confirmed via mass spectrometry that mice receiving systemic SPD treatment had an increase in SPD levels within the tumor microenvironment, recapitulating a high-SPD-producing tumor (Figure 1F). Additionally, systemic endogenous treatment with SPD robustly shortened survival of immune-competent mice (Figure 1, G and H, and Supplemental Figure 4, A–D). Taken together, these data suggest that SPD is elevated in the GBM microenvironment and accelerates GBM progression.

### SPD drives GBM growth in an immune-dependent manner.

**Figure 2:**
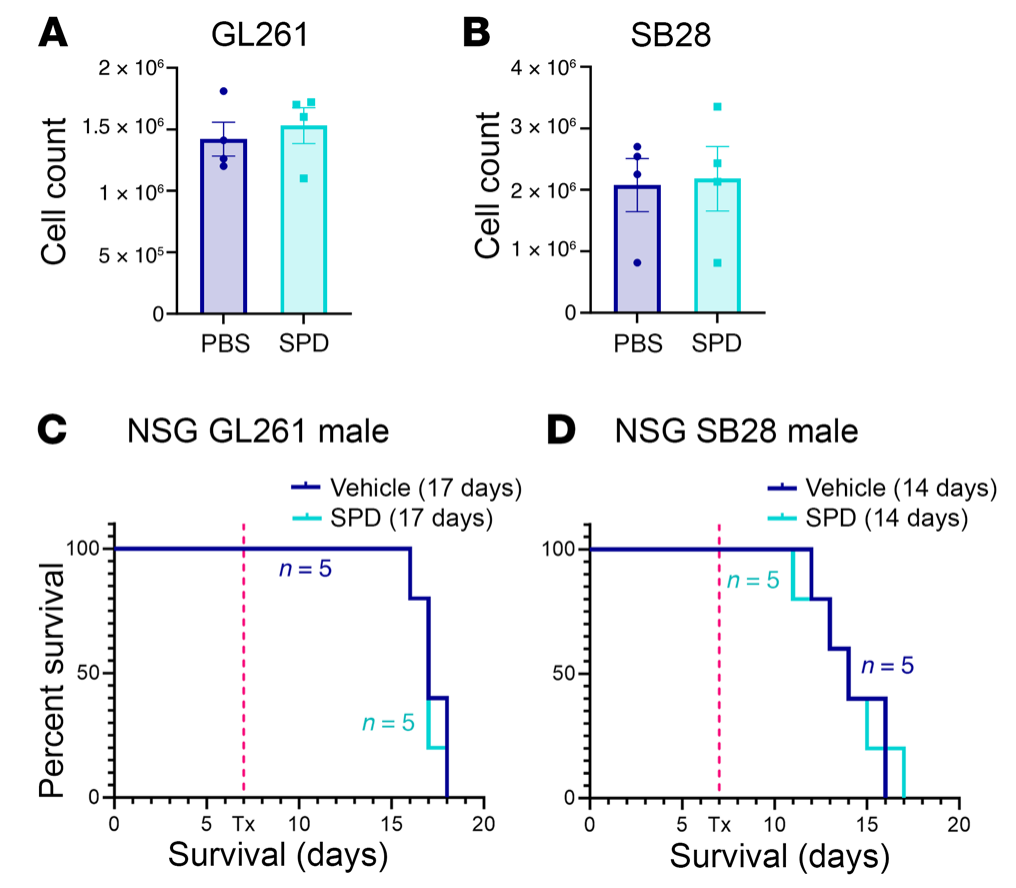
SPD interacts with the immune system to drive GBM progression. (**A** and **B**) Mouse glioma cells treated with 5 μM SPD in vitro for 72 hours; data are representative of 3 independent experiments. (**C** and **D**) Survival analysis was performed after intracranial injection of mouse GBM cell lines (25,000 GL261 cells per injection, 20,000 SB28 cells per injection) in immunocompromised male NSG mice, followed by 50 mg/kg SPD i.p. treatment or PBS vehicle. Median survival days and number of animals are indicated in the graphs. Statistical significance was determined by log-rank test, considering *P* values less than 0.05 to be significant.

As SPD is involved in many cellular functions, including cell growth, we tested whether SPD has a direct effect on cancer cell growth. When mouse glioma cells were cultured in vitro with SPD for 72 hours, we observed no significant changes in cell numbers in comparison with control treatment (Figure 2, A and B). Additionally, the proliferation rate of brain-resident populations (astrocytes, microglia) as well as human GBM and prostate cancer cells was not affected by the addition of exogenous SPD (Supplemental Figure 5, A–F). As SPD treatment did not directly increase tumor cell growth, we shifted our focus to other components of the tumor microenvironment that could contribute to the observed survival phenotype. GBM creates an immunosuppressive microenvironment characterized by an increase in immunosuppressive myeloid cells and limited T and NK cell infiltration (29, 30). Moreover, polyamines were recently shown to be critical for myeloid-driven immune suppression in GBM and T cell differentiation (11, 22, 31). To investigate whether SPD could be altering immune cells, we repeated the same in vivo experimental paradigm previously described (Figure 1E) using immunocompromised NSG (NOD.Cg-Prkdc^scid^Il2rg^tm1Wjl^/SzJ) mice. The sharp decline in survival observed in immune-competent mice with SPD treatment was abrogated in NSG mice, indicating that increased SPD was likely interfering with the immune response (Figure 2, C and D, and Supplemental Figure 6, A–D). These data suggest that SPD likely drives tumor growth in an immune cell–dependent manner.

### SPD drives GBM growth by reducing T cells.

**Figure 3:**
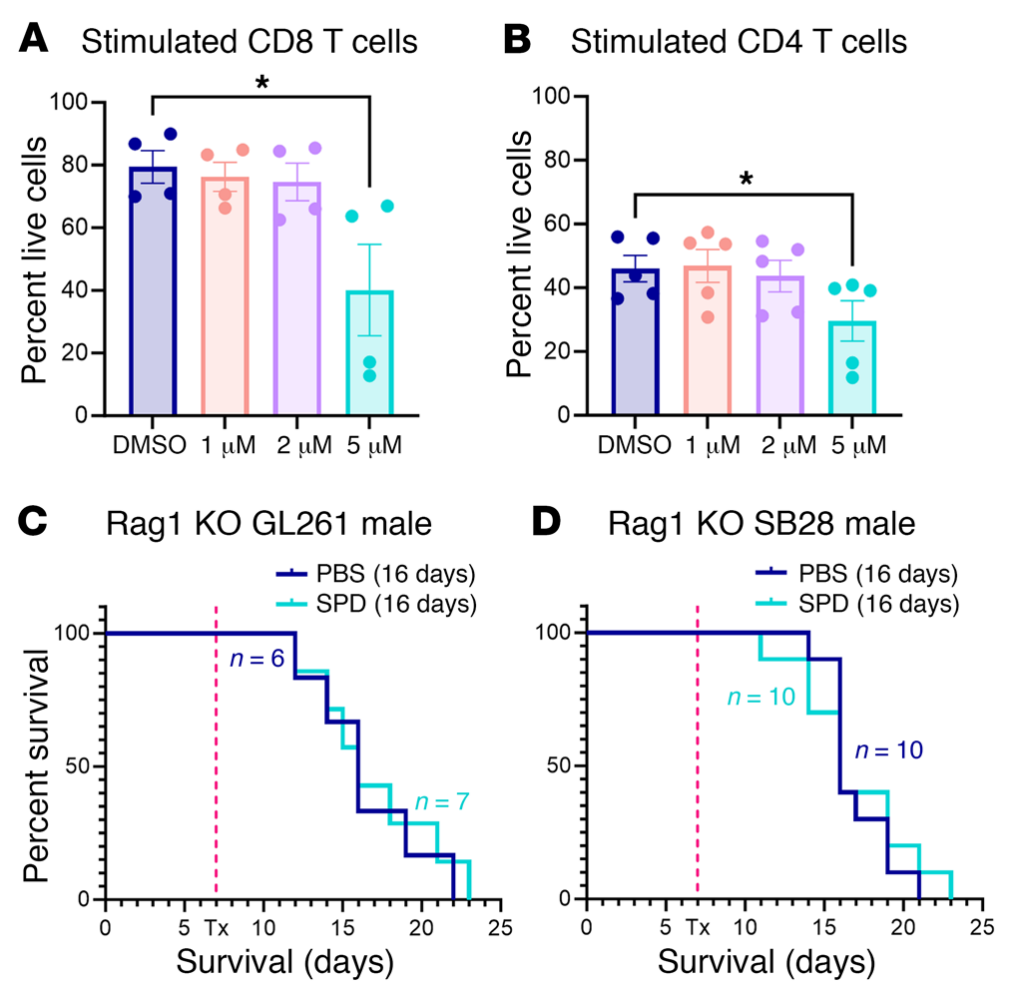
Lymphocyte subsets are affected by SPD. (**A** and **B**) Splenocyte-derived lymphocyte subsets were treated with physiological levels of SPD in vitro; data are representative of 3 independent experiments. (**C** and **D**) Survival analysis was performed after intracranial injection of mouse GBM cell lines (25,000 GL261 cells per injection, 20,000 SB28 cells per injection) in male Rag1-knockout mice, followed by 50 mg/kg SPD i.p. treatment or PBS vehicle. Median survival days and number of animals are indicated in the graphs. Data combined from 2 independent experiments. Statistical significance for **A** and **B** was determined by 1-way ANOVA (*P < 0.05). Statistical significance for **C** and **D** was determined by log-rank test, considering *P* values less than 0.05 to be significant.

Based on previous reports indicating that SPD drives CD4^+^ T cell differentiation (31), we investigated the effect of SPD on adaptive immune cells. Mouse splenocyte-derived lymphocytes treated with SPD in vitro and measured via flow cytometry showed a significant reduction in both viable CD8^+^ and CD4^+^ T cells (Figure 3, A and B), as well as in B cells and NK cells (Supplemental Figure 7, A–C). To determine whether lymphocytes were driving SPD-mediated accelerated GBM growth in our mouse models, we repeated the same experimental paradigm as described above (Figure 1E) in Rag1-knockout mice, which lack functional B and T cells. We observed no difference in survival between SPD and control treatment groups, supporting the hypothesis that SPD interacts with these immune cell subsets to drive GBM progression (Figure 3, C and D, and Supplemental Figure 7, D–G).

**Figure 4:**
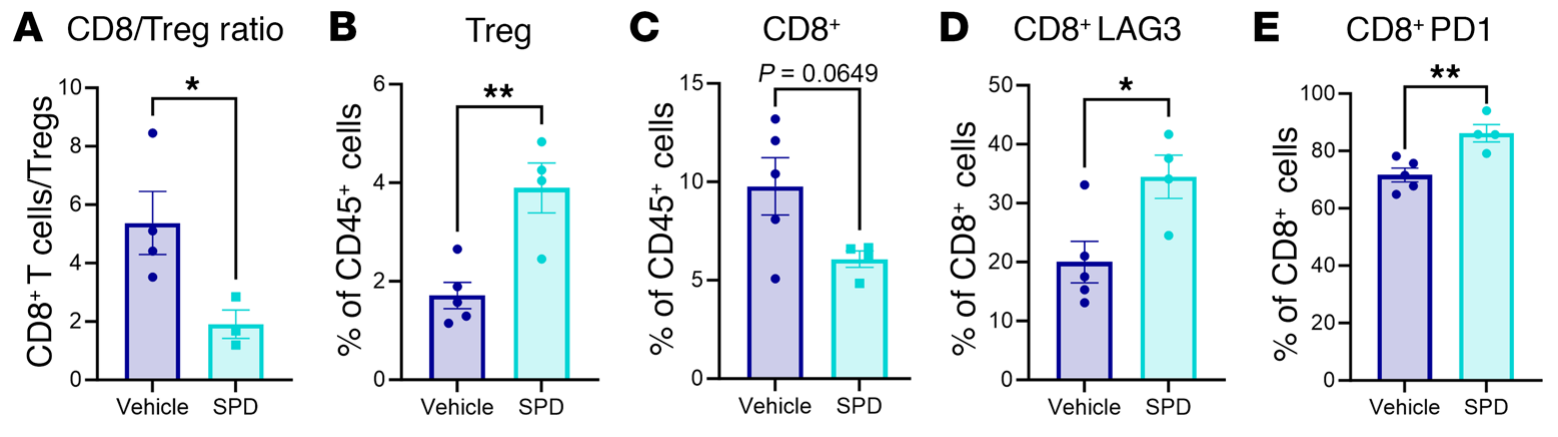
Exogenous treatment with SPD decreases cytotoxicity of CD8^+^ T cells. After intracranial injection of mouse GBM cell line SB28 (20,000 cells per injection) into male B6 mice followed by 50 mg/kg SPD i.p. treatment or PBS vehicle, the tumor-bearing hemisphere was collected and processed for flow cytometry immune phenotyping. (**A**) Ratio of CD8^+^ T cells to CD4^+^ Tregs. (**B** and **C**) Proportion of T cells in CD45^+^ cells. (**D** and **E**) Exhaustion markers of CD8^+^ T cells. Statistical significance for **A**–**E** was determined by unpaired, 2-tailed *t* test (*P < 0.05, **P < 0.01).

We then investigated changes to the immune response in the GBM microenvironment of immune-competent mice treated with exogenous SPD compared with control conditions. In the tumor-bearing hemisphere, we observed a significant reduction in the CD8^+^/Treg ratio, indicating decreased cytotoxic immune response in SPD-treated mice (Figure 4A). This was partially due to the increased proportion of Tregs and a trend of decreasing of CD8^+^ T cell abundance (Figure 4, B and C). Additionally, we observed increased exhaustion markers specifically on CD8^+^ T cells in SPD-treated mice (Figure 4, D and E). Immune analysis of blood and bone marrow replicated the immunosuppressive phenotype seen in the tumor tissue (Supplemental Figure 8, A–I). Treg exhaustion markers were not affected by SPD treatment (Supplemental Figure 8, J–M). Immune phenotyping of tumor-bearing mice suggested that increased SPD levels in the tumor microenvironment affected CD8^+^ T cells and Tregs, contributing to GBM progression (representative gating strategy in Supplemental Figure 9). Taken together, these data demonstrate that SPD reduces cytotoxic T cell number and phenotype.

### Ornithine decarboxylase drives GBM cell–mediated tumor growth and T cell alterations.

**Figure 5:**
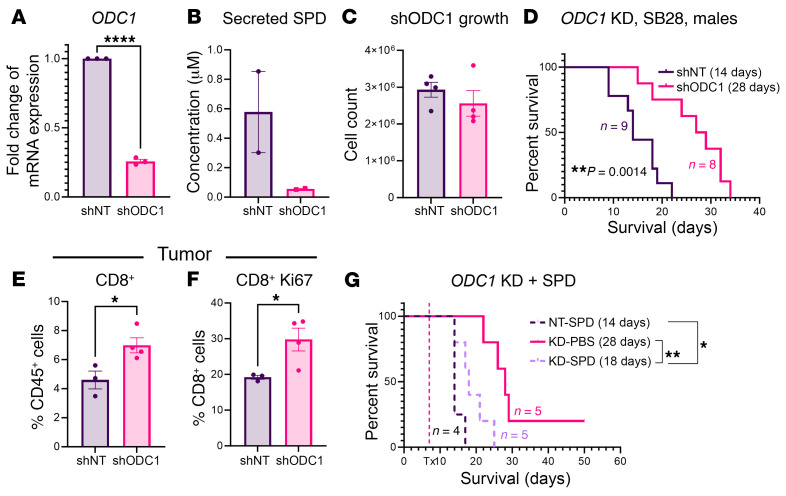
Knockdown of the polyamine biosynthesis pathway extends survival. (**A**) mRNA expression of *ODC1* in shRNA knockdown mouse glioma cells compared with non-targeted control. (**B**) Conditioned medium SPD measurement via mass spectrometry. (**C**) Cell count after 72 hours of growth. (**D**) Survival analysis was performed after intracranial injection of shRNA-modified mouse GBM cells (20,000 non-target or *ODC1*-knockdown [KD] SB28 cells) in B6 mice. Median survival days and number of animals are indicated in the graph. Data combined from 2 independent experiments. (**E** and **F**) Immune phenotyping via flow cytometry was performed on tumors removed from B6 mice 14 days after intracranial injection of shRNA-modified mouse GBM cells (20,000 non-target or *ODC1-*KD SB28 cells). (**E**) Percentage of CD8^+^ cells in tumor. (**F**) Proliferation marker in CD8^+^ T cells. (**G**) Survival analysis was performed after intracranial injection of shRNA-modified mouse GBM cells (20,000 non-target or *ODC1-*KD SB28 cells) in B6 mice, followed by SPD or PBS vehicle treatment as described in Figure 1E. Median survival days and number of animals are indicated on the graph. Statistical significance for **D** and **G** was determined by log-rank test, considering *P* values less than 0.05 to be significant (*P < 0.05, **P < 0.01). Statistical significance for **A**, **C**, **E**, and **F** was determined by unpaired, 2-tailed *t* test (*P < 0.05, ****P < 0.0001).

Given that exogenously administered SPD drives tumor growth and alters T cell number and phenotype, we wanted to assess how this functions in a GBM cell–intrinsic manner. Using shRNA lentiviral particles targeting *ODC1*, the gene that encodes ornithine decarboxylase (ODC) — the rate-limiting irreversible enzyme of the main polyamine biosynthesis pathway — we knocked down *ODC1* in SB28 tumor cells (Figure 5A), which resulted in decreased SPD production (Figure 5B) and no significant changes in intrinsic tumor cell growth (Figure 5C). Intracranial implantation of *ODC1*-knockdown GBM cells resulted in significantly extended survival compared with a non-target control (Figure 5D), indicating that SPD production by cancer cells is partially responsible for GBM growth. Immune phenotyping of mice implanted with *ODC1*-knockdown cells revealed an increase in the proportion of CD8^+^ T cells in the tumor microenvironment in comparison with non-targeting controls (Figure 5E). Additionally, the CD8^+^ T cell proliferation marker Ki-67 was increased, suggesting that the CD8^+^ T cells might have increased expansion in the tumor microenvironment (Figure 5F). To investigate how specifically this result was due to SPD itself, we repeated the original exogenous SPD administration paradigm (Figure 1E) in mice with *ODC1-*knockdown cells. When mice bearing *ODC1-*knockdown tumors were treated with systemic SPD, we observed a partial rescue of our original phenotype, indicating that tumor cell–derived SPD is a significant contributor of GBM progression (Figure 5G). Together, these data suggest that SPD generated by GBM cells via ODC can drive GBM growth and attenuate T cell number and function, which is consistent with our findings observed with exogenous administration of SPD.

### SPD induces CD8^+^ T cell apoptosis and decreases functionality.

**Figure 6:**
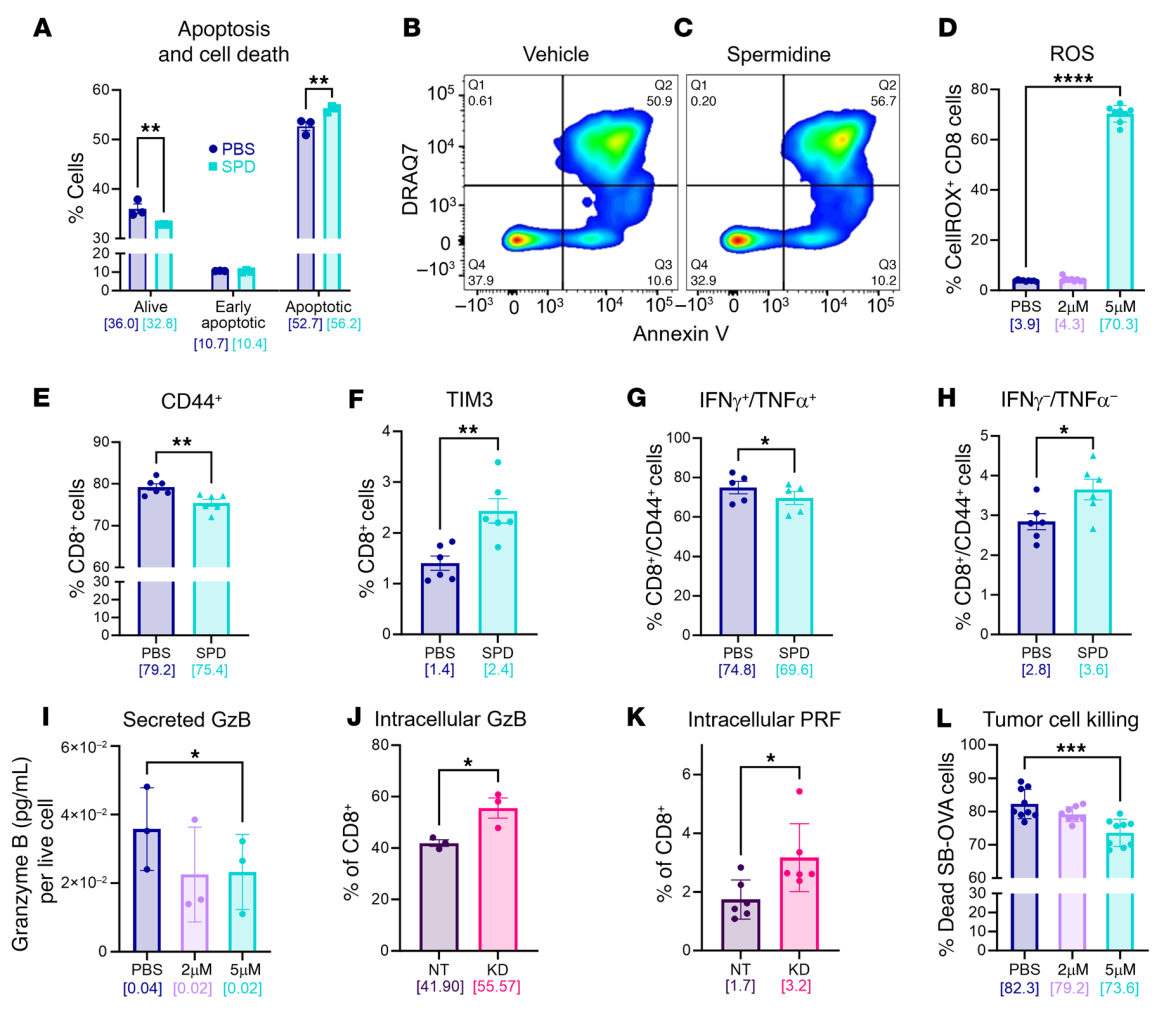
CD8^+^ T cells have reduced viability and functionality in the presence of SPD. (**A**–**C**) Splenocyte-derived CD8^+^ T cells were treated with 5 μM SPD in vitro. (**A**) Apoptotic cells and cell death were measured via annexin V and DRAQ7 staining, respectively, and analyzed via flow cytometry; data are representative of 3 independent experiments. (**B** and **C**) Visual representation of gain in double-positive cells under SPD treatment. (**D**) ROS levels in CD8^+^ T cells treated with varying concentrations of SPD measured via CellROX flow cytometry assay; data are representative of 3 independent experiments. (**E** and **F**) T cell markers in CD8^+^ population treated with PBS or 5 μM SPD. (**G** and **H**) IFN-γ^+^TNF-α^+^ (**G**) and IFN-γ^–^TNF-α^–^ (**H**) subsets in the CD8^+^CD44^+^ T cell population. (**I**) Granzyme B (GzB) levels measured via ELISA in conditioned medium from CD8^+^ T cells treated in vitro with varying concentrations of SPD; data are representative of 3 independent experiments. (**J** and **K**) Intracellular flow cytometry measurement of GzB (**J**) and perforin (PRF) (**K**) in CD8^+^ T cells treated with conditioned medium from non-target or ODC1-KD cells; data are representative of 3 independent experiments. (**L**) Viability of tumor cells after Transwell coculture with SPD-treated CD8^+^ T cells via cell killing assay; data combined from 3 experiments. Statistical significance in **A** was determined by 2-way ANOVA (**P < 0.01). Statistical significance in **D**, **I**, and **L** was determined by 1-way ANOVA (*P < 0.05, ***P < 0.001, ****P < 0.0001). Statistical significance in **E**–**H**, **J**, and **K** was determined by unpaired, 2-tailed *t* test (*P < 0.05, **P < 0.01). Bracketed numbers indicate the mean.

To elucidate the mechanism through which SPD affects CD8^+^ T cells, we first investigated cell death and apoptosis, as SPD is known to be involved in apoptotic pathways (32). Treating splenocyte-derived CD8^+^ T cells with SPD during the in vitro stimulation process for 72 hours resulted in an increase in fully apoptotic cells and a reduction in live cells (Figure 6, A–C). Additionally, the death of CD8^+^ T cells can partially be attributed to increased reactive oxygen species (ROS) after treatment with SPD during stimulation (Figure 6D). No difference was noted in cell proliferation of CD8^+^ T cells treated with SPD compared with vehicle-treated cells (Supplemental Figure 10A). CD8^+^ T cells treated in the same manner were analyzed for cytokine profile changes; we observed an increase in the exhaustion marker TIM3 as well as a reduction of the activation marker CD44 (Figure 6, E and F). The number of CD8^+^ T cells producing the established anti-tumorigenic cytokines IFN-γ and TNF-α was reduced (Figure 6, G and H). Investigating functional protease granzyme B (GzB) in the same treated CD8^+^ T cells revealed a decrease in secreted GzB per live cell (Figure 6I), indicating a reduction of functionality of CD8^+^ T cells treated with SPD. Additionally, when exposing CD8^+^ T cells to conditioned medium collected from *ODC1*-knockdown cells, we observed an increase in both GzB and perforin, suggesting that tumor-derived polyamines affect functionality of CD8^+^ T cells (Figure 6, J and K). To explore the full effect of these changes in secreted cytotoxic and inflammatory molecules, we used a tumor cell killing assay to assess changes in cell death. OT-I CD8^+^ T cells were pretreated with PBS control or varying concentrations of SPD, then added in a Transwell to coculture with previously plated SB28 cells overexpressing ovalbumin (SB28-OVA cells). Viability of the tumor cells measured via flow cytometry showed a reduced ability of CD8^+^ T cells to kill tumor cells in a concentration-dependent manner (Figure 6L). Taken together, these data suggest that SPD increases apoptosis and ROS, thus decreasing the available cytotoxic cells in the CD8^+^ T cell pool, in addition to decreasing their killing functionality by altering their cytokine profile and inflammatory phenotype.

### SPD is correlated with decreased CD8^+^ T cells and a poorer prognosis.

**Figure 7:**
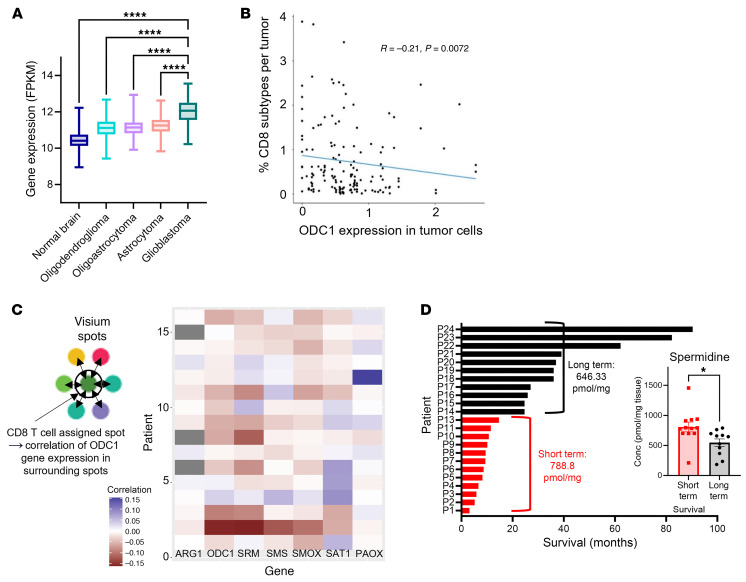
Patients with GBM have increased *ODC1* expression and high SPD levels that are correlated with poorer prognosis. (**A**) mRNA expression of *ODC1* from GTEX non-neoplastic and TCGA lower-grade gliomas and GBM tumor tissue, as notated in 2011 WHO classification. FPKM, fragments per kilobase of transcript per million mapped reads. (**B**) Single-cell RNA-Seq correlation of *ODC1* expression in tumor cells and number of CD8^+^ cells in the tumor microenvironment. (**C**) Schematic of Visium single-cell analysis; heatmap showing that presence of CD8^+^ T cells correlates with surrounding polyamine pathway gene expression by tumor cells. ARG1, arginase 1; ODC1, ornithine decarboxylase; PAOX, polyamine oxidase; SAT1, spermidine/spermine acetyltransferase 1; SMOX, spermidine oxidase; SRM, spermidine synthase. (**D**) Long-term versus short-term survivor SPD levels in tumor tissue at primary resection of patients with GBM; metabolites measured via liquid chromatography–tandem mass spectrometry. Statistical significance in **A** was determined by 1-way ANOVA (****P < 0.001). Statistical significance in **B** was determined by linear regression. Statistical significance in **D** was determined by unpaired, 2-tailed *t* test (*P < 0.05).

To investigate parallels between patients with GBM and our preclinical findings, we interrogated multiple components of the SPD pathway and the tumor microenvironment. The Cancer Genome Atlas (TCGA) and Genotype-Tissue Expression (GTEx) data on normal brain tissue compared with low-grade glioma showed an increase in *ODC1* mRNA expression; there was a robust increase in expression in patients with GBM compared with all other groups (Figure 7A). To assess whether *ODC1* expression is linked to changes in the immune microenvironment, we analyzed single-cell RNA-Seq data from Ruiz-Moreno et al. (33) and found that higher expression of *ODC1* in cancer cells correlated with fewer CD8^+^ T cells in the tumor microenvironment in patients with GBM (Figure 7B), similar to what we observed in mouse models. Furthermore, Visium spatial analysis (Visium Technologies) of patients with GBM from the work by Ravi et al. (34) showed a negative correlation between SPD-producing enzymes and the areas immediately surrounding identified CD8^+^ T cells (Figure 7C). Finally, to link SPD levels to GBM patient survival, tumor samples from age-matched patients with GBM were analyzed via liquid chromatography–tandem mass spectrometry. Short-term survivors (median survival 9.8 months) had significantly higher levels of SPD in their tumors at primary resection than long-term survivors (median survival 36.03 months) (Figure 7D). Additionally, patients in the lowest quartile of SPD levels survived much longer compared with the highest quartile, indicating there is a negative correlation between intratumoral SPD levels and overall survival (Supplemental Figure 11A). Additional members of the polyamine family were not as strongly correlative in quartile testing; however, we did see similar trends when survival above and below the median was analyzed (Supplemental Figure 11, B–F). Taken together, these data further reinforce that SPD is associated with poor GBM patient outcome and a reduction in CD8^+^ T cells in the tumor microenvironment.

## Discussion

Here, we identify a new molecular mechanism through which GBM cells affect their surrounding microenvironment and drive a protumorigenic state through direct depletion and impairment of T cells. This immune alteration occurs via increased SPD in the tumor microenvironment and is driven by expression of ODC, the rate-limiting enzyme in the main polyamine biosynthesis pathway. These findings reinforce a model in which tumor cells secrete a host of factors to alter the immune microenvironment in their favor. Our findings show that SPD itself, either increased via exogenous addition or reduced via *ODC1* knockdown, did not alter intrinsic tumor growth but did impact cytotoxic T cells and Tregs. These results are similar to our previous observation in which GBM cancer stem cells secreted macrophage migration inhibitory factor, which supported myeloid-derived suppressor cell function but was dispensable for tumor cell growth (35). It is worth noting that other studies have demonstrated an essential role for SPD in tumor cell growth, including in pediatric glioma and neuroblastoma. With respect to the differences between GBM and pediatric glioma in terms of SPD dependency, this could be due to inherent mutational landscapes and/or differential metabolic dependencies. Another possibility could be differing SPD levels between pediatric glioma and GBM cells, as previous observations in pediatric glioma were not directly compared with GBM models. It could be the case that GBM cells have an increased level of SPD at baseline compared with pediatric glioma cells; in this case, increasing SPD would not elicit a progrowth phenotype, and knockdown, which we used here instead of complete knockout, would maintain a sufficient amount of SPD present to perpetuate cell growth.

Our data support a model in which CD8^+^ T cells in the GBM microenvironment are more sensitive to changes in SPD compared with other immune cells. These findings are complementary to recent work in tumor-associated myeloid cells and may help explain why SPD generates a protumorigenic environment (22), as it can increase immune suppression through enhancement of myeloid cells while concomitantly decreasing immune activation though the depletion and reduced cytokine production among CD8^+^ T cells. Future studies would benefit from direct comparison between these two protumorigenic mechanisms to determine which population is more responsive to SPD, either directly or through other immune alterations.

While our studies focused on SPD, the polyamine family also contains the additional metabolites putrescine and spermine, as well as cadaverine, which is produced solely by bacteria. We observed that exogenous spermine administration does, to an extent, replicate the effects of SPD administration, resulting in a shortening of survival (data not shown). There could be several reasons for the specificity of SPD compared with other polyamine family members. Although some polyamine functions are shared by all members, certain functions are driven mostly by a particular polyamine compared with the others. Cell necrosis and apoptosis are mediated by putrescine and SPD (20). Another function that is more specific to SPD is inflammation reduction (36, 37). This correlates with the immune suppression we see in our studies as well as the characterization of GBM as a “cold tumor” (38). While our studies focused on GBM, polyamines have been reported to have a protumorigenic role in established tumors in other cancers — such as prostate and colorectal — and a tumor-suppressive role at the initial stages in other tumors — namely melanoma and some types of breast cancer (39–43). Therefore, our findings may be of interest for other tumor types.

Our studies leverage preclinical models to demonstrate that SPD can drive tumor growth in an immune-dependent manner and are consistent with other pediatric and adult brain tumor preclinical findings. Conceptually, these findings support the use of polyamine inhibitors for malignant brain tumors. However, current attempts to target these pathways via difluoromethylornithine, which is decarboxylated by ODC and binds to the enzyme, thus irreversibly inactivating it, have shown modest clinical efficacy (44). This could partially be due to the ubiquitous nature of polyamines in the human body. Although this inhibitor blocks de novo biosynthesis of polyamines, uptake of polyamines secreted by other cells in the environment could help maintain tumor cell growth and sustain pressure on the immune response. While our studies focus on the function of tumor cell–derived SPD in altering the immune microenvironment, how SPD is transported into cells was not assessed. SPD can be taken into cells via a known polyamine transporter, SLC3A2, which we found to be expressed in multiple immune lineages using human single-cell RNA-Seq data (GBmap; Ruiz-Moreno et al., ref. 33), and we confirmed similar expression between mouse myeloid (CD11b^+^), CD4^+^, and CD8^+^ T cells (data not shown). These observations suggest that immune cells express the relevant polyamine transporter, and future studies could focus on the function of these transporters in immune cells. Additional studies could investigate the consequence of targeting SLC3A2, including the use of available inhibitors in combination with the polyamine pathway inhibitor difluoromethylornithine. Successfully targeting the polyamine pathway will most likely require combination intervention at multiple enzyme steps in addition to transport inhibitors. Blocking both ODC and spermidine synthase (SRM) would provide a more complete elimination of SPD by interfering with both de novo synthesis from ornithine and from a putrescine precursor; however, a reliable inhibitor of SRM remains elusive at this point.

We should note that there are also limitations to our current study. The majority of our assessments are based in mouse models, and while we have some indication that SPD may function in humans in a manner similar to that in our preclinical models, additional interrogation of SPD and other polyamines in human tissue, cerebrospinal fluid, and blood regarding tumor progression across a large cohort would be useful to determine the extent to which elevated SPD levels indicate immune suppression and poor prognosis. Though our studies leveraged mouse models for the assessment of ODC function, we found that *ODC1* expression was present across human GBM tumors, irrespective of tumor subtypes/states (data not shown). While our studies focused on lymphocyte changes, there are reports of a contribution by myeloid cells (22, 45, 46), and together, these immune cell types could synergistically create a more protumorigenic microenvironment. Focused studies interrogating both myeloid and lymphoid components will help clarify the effect of SPD on each immune lineage. As there is not one clear mechanism that accounts for the majority of cytotoxic T cell depletion and loss of functionality in an SPD-dependent manner, additional clarification is required to facilitate targeting strategies. Notably, while polyamines have been shown to impact T cell lineage specification via hypusination (47), we did not observe an increase in hypusination in bone marrow–derived cells treated with SPD (data not shown), which could be due to many factors, including alternative pathway utilization. Other proposed mechanisms of action in which SPD plays a role, such as T cell receptor clustering and epigenetic alterations, need to be studied to provide a more complete picture of how CD8^+^ T cells are affected by SPD in the tumor microenvironment. Finally, as our studies focused on polyamines produced by GBM cells in the tumor microenvironment, it should be noted that peripheral polyamines, including those originating from the gut microbiome, could also play a role in the overall immune response to GBM.

Our observations support a role for SPD in the tumor microenvironment driving tumor growth, but there are also several unanswered questions based on these initial findings. We know that tumor cells produce higher levels of polyamines, but polyamines are also produced by other cells in the body and commensal gut microbes and are also taken in as part of the diet. Gut dysbiosis has been noted in many cancers, including GBM (48, 49), and there is a possibility that microbial reorganization in the gut could become skewed toward polyamine-producing strains, which would result in an increase in polyamines in the circulation and tumor microenvironment, thereby inducing immune suppression. Additionally, standard of care for GBM (surgical resection, radiation, chemotherapy) could affect both cellular production of polyamines and the gut microbiome, and this could result in alteration of the pool of polyamines or polyamine precursors available to cells in the tumor microenvironment. We show a reduction of cytotoxic immune response partially due to a reduction in CD8^+^ T cells and an increase in Tregs. The majority of immunotherapies rely on the presence of CD8^+^ T cells in the tumor microenvironment in order to augment their exhaustion and activation profiles (50). Potentially, the inhibition of polyamine synthesis combined with the introduction of immunotherapies such as checkpoint inhibitors could increase the efficacy of immunotherapy in GBM. Finally, sex differences in the immune response have been noted in GBM, not only in localization of immune cells but also in their function and response to immunotherapies (26, 51), and the extent to which SPD and polyamines function in the context of sex differences is unclear. In our preclinical studies, we assessed males and females and observed no substantial sex differences; however, future therapeutic studies should consider sex as a biological variable given the above-mentioned reports. Taken together, our data highlight the communication between tumor cells and immune cells, which results in a favorable immune microenvironment for GBM growth and provides a function for SPD in the tumor microenvironment in facilitating this process.

## Methods

### Sex as a biological variable

Our study examined male and female animals, and similar findings are reported for both sexes.

### Cell models

The syngeneic mouse GBM cell model SB28 and SB28-OVA were a gift from Hideho Okada (UCSF), and GL261 cells were obtained from the Developmental Therapeutic Program at the National Cancer Institute. The CT-2A cell model was a gift from Misty Jenkins at the Walter and Eliza Hall Institute of Medical Research (Melbourne, Australia). PC-3 human prostate cancer cells were obtained from the Cleveland Clinic Lerner Research Institute. The patient-derived GBM model DI318 was derived at the Cleveland Clinic Lerner Research Institute, L1 was obtained from the University of Florida (Gainesville, Florida, USA), and 3832 was obtained from Duke University (Durham, North Carolina, USA). Human astrocytes were purchased from ScienCell. All cell lines were treated with 1:100 MycoRemoval Agent (MP Biomedicals) upon thawing and routinely tested for *Mycoplasma* spp. (Lonza). Mouse GBM cell lines and human prostate cancer cells were maintained in complete RPMI 1640 (Media Preparation Core, Cleveland Clinic) supplemented with 10% FBS (Thermo Fisher Scientific) and 1% penicillin/streptomycin (Media Preparation Core, Cleveland Clinic). Human GBM lines, human astrocytes, and primary mouse microglia and astrocytes were maintained in complete DMEM:F12 (Media Preparation Core, Cleveland Clinic) supplemented with 1% penicillin/streptomycin, 1× N-2 Supplement (Gibco), and EGF/FGF-2. All cells were maintained in humidified incubators held at 37°C and 5% CO_2_ and not grown for more than 20 passages.

### Mice

All animal procedures were performed in accordance with the guidelines and protocols approved by the Institutional Animal Care and Use Committee at the Cleveland Clinic and by the Walter and Eliza Hall Institute Animal Ethics Committee. *C57BL/6* (RRID:IMSR_JAX:000664), *RAG1^–/–^* (B6.129S7-Rag1tm1Mom/J; RRID:IMSR_JAX:002216), and *OT-I TCR* transgenic [C57BL/6-Tg(TcraTcrb)1100Mjb/J; RRID:IMSR_JAX:003831] male and female mice (4–12 weeks of age) were purchased from The Jackson Laboratory as required. *NSG* (NOD.Cg-Prkdc^scid^Il2rg^tm1Wjl^/SzJ) mice were obtained from the Biological Research Unit (BRU) at Lerner Research Institute, Cleveland Clinic. All animals were housed in a specific pathogen–free facility of the Cleveland Clinic BRU with a 12-hour light/12-hour dark cycle. All animals were maintained on a control diet to minimize/normalize polyamines consumed via the diet (Research Diets, D12450J).

For tumor implantation, 5- to 8-week-old mice were anesthetized, fit to a stereotactic apparatus, and intracranially injected with 10,000–25,000 tumor cells in 5 μL RPMI-null medium into the left hemisphere approximately 0.5 mm rostral and 1.8 mm lateral to the bregma with 3.5 mm depth from the scalp. In CT-2A experiments, 10,000 tumor cells were injected 1 mm lateral, 1 mm anterior with 2.5 mm depth. In some experiments, 5 μL null medium was injected into age- and sex-matched animals for sham controls. Animals were monitored over time for the presentation of neurological and behavioral symptoms associated with the presence of a brain tumor. Biological sex is indicated for each study.

In some experiments, mice were treated with 50 mg/kg SPD (MilliporeSigma, catalog S0266) diluted in 0.9% saline or 0.9% saline control intraperitoneally starting from 7 days after tumor implantation; mice received 3 injections per week until endpoint.

### Isolation of ex vivo mouse cells for in vitro testing

#### Microglia and astrocytes.

Primary mouse microglia and astrocytes were isolated and cultured from day 0–1 wild-type B6 pup brains, as previously described (52).

#### CD8^+^CD4^+^ T cells and Tregs.

CD8^+^CD4^+^ T cells were isolated from splenocytes of 8- to 12-week-old mice using magnetic bead isolation kits (Stemcell Technologies). Isolated CD8^+^ T cells were cultured in the presence of recombinant human IL-2 (100 U/mL; PeproTech) and anti-CD3/CD28 Dynabeads (Thermo Fisher Scientific) for 3–4 days before flow cytometry studies. T regulatory cells (Tregs) were cultured from CD4^+^ T cells and induced with IL-2 (100 U/mL; PeproTech), anti-CD3/CD28 Dynabeads (Thermo Fisher Scientific), and TGF-β (5 ng/mL; PeproTech). For proliferation studies, T cells were stained with 1:1,000 CellTrace Violet (Invitrogen) before culturing.

#### Myeloid-derived suppressor cells.

Bone marrow was isolated from the femur and tibia of 8- to 12-week-old mice. Two million bone marrow cells were cultured in 6-well plates in 2 mL RPMI/10% FBS supplemented with 40 ng/mL GM-CSF and 80 ng/mL IL-13 (PeproTech) for 3–4 days. Cells were stained for viability, blocked with Fc receptor inhibitor, and stained with a combination of CD11b, Ly6C, and Ly6G for sorting of myeloid-derived suppressor cell (MDSC) subsets (monocytic MDSCs, CD11b^+^Ly6C^+^Ly6G^−^, vs. granulocytic MDSCs, CD11b^+^Ly6C^−^Ly6G^+^) and the control population (CD11b^+^Ly6C^−^Ly6G^−^) using a BD FACSAria II (BD Biosciences).

### Cell viability and functionality assays

The cell models described above were treated with varying concentrations of SPD in DMSO/PBS or equivalent vehicle in respective complete media. At the time points described in the corresponding figure legends, single-cell suspensions were combined with an equal volume of 0.4% Trypan Blue (Thermo Fisher Scientific) and counted using a TC20 Automatic Cell Counter (Bio-Rad). Alternatively, an equal volume of CellTiter-Glo Luminescent Cell Viability Assay (Promega) was added to treated cells, and viability was measured via luminescence on a VICTOR Nivo multimode plate reader (PerkinElmer).

To measure cell death and apoptosis of CD8^+^ T cells treated in vitro with SPD, FITC-labeled annexin V (BioLegend) and DRAQ7 (Invitrogen) were added in accordance with the manufacturer’s protocols. To measure intracellular pH levels, CD8^+^ T cells were labeled with pHrodo Red (Thermo Fisher Scientific) according to the manufacturer’s protocol. Samples were run on an LSR Fortessa flow cytometer (BD Biosciences) with a minimum of 10,000 events collected. Single cells were gated, and the percentages of annexin V–positive and/or DRAQ7-positive cells were determined. For pHrodo Red–labeled cells, high and low gates were used to determine intracellular acidic and neutral pH based on geometric mean fluorescence intensity (a measure of the shift in fluorescence intensity of a population of cells). For intracellular cytokine detection, cells were stimulated using Cell Stimulation Cocktail plus protein transport inhibitor (eBioscience) in complete RPMI for 4 hours. After stimulation, cells were subjected to the flow cytometry staining procedures described below. To investigate any changes in ROS levels, isolated CD8^+^ T cells were treated with varying concentrations of SPD in vitro, and then ROS was measured by dark red CellROX assay (Thermo Fisher Scientific) according to the manufacturer’s protocol and analyzed on an LSR Fortessa flow cytometer.

### Transwell coculture cell killing assessment by flow cytometry

SB28-OVA mouse GBM cells were plated in tissue culture wells. CD8^+^ T cells were isolated from splenocytes of OT-I mice and activated with ovalbumin peptide fragment 323–339 in the presence of varying concentrations of SPD for 3 days. A 2:1 ratio of CD8^+^ T cells to SB28-OVA GBM cells was plated in a Transwell insert (5-μm pore size; Corning), which was then submerged in the culture medium of the underlying culture well. Transwell experiments were analyzed on a BD LSR Fortessa (BD Biosciences) operated by BD FACSDiva software (v9.0). FlowJo software (BD Biosciences,10.8.1) was used to analyze flow cytometry data.

### Granzyme B enzyme-linked immunosorbent assay

Levels of granzyme B secreted into conditioned medium were measured using the Mouse Granzyme B ELISA SimpleStep kit (Abcam) following the manufacturer’s protocols.

### Liquid chromatography–mass spectrometry quantification of polyamine metabolites

#### Sample preparation.

Plasma and tissue samples for polyamine quantitation were processed as previously described for serum samples, with minor modifications as below (53).

Twenty microliters of plasma was aliquoted into a 12 × 75 mm glass tube and mixed with 5 μL internal standard mix consisting of [^2^H_5_]ornithine, [^13^C_6_]arginine, [^2^H_8_]spermine, [^2^H_8_]spermidine, [^13^C_4_]putrescine, and [^2^H_3_]acisoga in water with a concentration (in μM) of 400, 400, 10, 10, 10, and 0.5, respectively. Then 5 μL of 1 M sodium carbonate (pH 9.0) and 10 μL isobutyl chloroformate were added to derivatize polyamines. Then 0.5 mL diethyl ether was added to extract the derivatized product. All the stable isotope internal standards were purchased from Cambridge Isotope Laboratories or CDN Isotopes.

For the tissue samples, approximately 20 mg brain tissue was mixed with 5 μL of the above internal standard mix in a 2 mL Eppendorf tube with 400 μL H_2_O, followed by homogenization in a tissue homogenizer (QIAGEN) with a metal bead (QIAGEN, 69997) added. The homogenate was spun down at 20,000*g* at 4°C for 10 minutes. Supernatant (200 μL) was transferred to a clean 12 × 75 mm glass tube, and 50 μL of 1 M sodium carbonate (pH 9.0) and 100 μL isobutyl chloroformate were added to derivatize polyamines. Then 2 mL diethyl ether was added to extract the derivatized product. The diethyl ether extract was dried under N_2_ and resuspended in 50 μL of 1:1 0.2% acetic acid in water/0.2% acetic acid in acetonitrile and transferred to a mass spectrometer with plastic insert for liquid chromatography–mass spectrometry assay.

#### Liquid chromatography–mass spectrometry assay.

Supernatants (5 μL) were analyzed by injection onto a Cadenza CD-C18 Column (50 × 2 mm; Imtaket) at a flow rate of 0.4 mL/min using a Vanquish LC autosampler interfaced with a Quantiva mass spectrometer (both from Thermo Fisher Scientific). A discontinuous gradient was generated to resolve the analytes by mixing of solvent A (0.2% acetic acid in water) with solvent B (0.2% acetic acid in acetonitrile) at different ratios starting from 0% B to 100% B. The mass parameters were optimized by injection of individual derivatized standard or isotope-labeled internal standard individually. Nitrogen (99.95% purity) was used as the source, and argon was used as collision gas. Various concentrations of non-isotopically-labeled polyamine standard mixed with internal standard mix undergoing the same sample procedure were used to prepare calibration curves.

### Immunophenotyping by flow cytometry

At the indicated time points, a single-cell suspension was prepared from the tumor-bearing left hemisphere by enzymatic digestion using collagenase IV (MilliporeSigma) and DNase I MilliporeSigma). Digested tissue was filtered through a 70 μm cell strainer, and lymphocytes were enriched by gradient centrifugation using 30% Percoll solution (MilliporeSigma). Cells were then filtered again with a 40 μm filter. Cells were stained with LIVE/DEAD Fixable stains (Thermo Fisher Scientific) on ice for 15 minutes. After washing with PBS, cells were resuspended in Fc receptor blocker (Miltenyi Biotec) diluted in PBS/2% BSA and incubated on ice for 10 minutes. For surface staining, fluorochrome-conjugated antibodies were diluted in Brilliant Buffer (BD) at 1:100–1:250, and cells were incubated on ice for 30 minutes. After washing with PBS/2% BSA buffer, cells were then fixed with FOXP3/Transcription Factor fixation buffer (eBioscience) overnight. For intracellular staining, antibodies were diluted in FOXP3/Transcription Factor permeabilization buffer at 1:250–1:500, and cells were incubated at room temperature for 45 minutes. For intracellular cytokine detection, cells were stimulated using Cell Stimulation Cocktail plus protein transport inhibitor (eBioscience) in complete RPMI for 4 hours. After stimulation, cells were subjected to the staining procedures described above. Stained cells were acquired with a BD LSR Fortessa or Aurora (Cytek) and analyzed using FlowJo software (v10, BD Biosciences).

### Reagents

For immunophenotyping in mouse models, the following fluorophore-conjugated antibodies at concentrations of 1:250–1:500 were used: CD11b (M1/70, catalog 563553), CD11c (HL3, catalog 612796, RRID:AB_2870123), CD3 (145-2C11, catalog 564379, RRID:AB_2738780), and CD44 (IM7, catalog 612799, RRID:AB_2870126) were obtained from BD Biosciences. CTLA4 (UC10-4B9, catalog 106312), PD1 (29F.1A12, catalog 135241), B220 (RA3-6B2, catalog 103237), Ki-67 (11Fb, catalog 151215), TIM3 (RMT3-23, catalog 119727), I-A/I-E (M5/114.15.2, catalog 107606), CD45 (30-F11, catalog 103132), LAG3 (C9B7W, catalog 125224), NK1.1 (PK136, catalog 108716), CD4 (GK1.5, catalog 100422), CD8 (6206.7, catalog 100712), granzyme B (QA18A28, catalog 396413), TNF-α (MP6-XT22, catalog 506329), and IFN-γ (XMG1.2, catalog 505846) were obtained from BioLegend. Anti-FOXP3 (FJK-16s, catalog 12-5773, RRID:AB_465936) antibody was obtained from eBioscience.

### Stable transduction with lentiviral shRNA

Lentifect Ultra-Purified Lentiviral Particles targeting mouse *ODC1* and an associated non-targeted control lentiviral particle were purchased from Genecopoiea. Before transfection, mouse glioma cells were grown to about 70% confluence on tissue culture–treated plates. Lentivirus was added to and incubated with the cells for 24 hours, followed by a change to fresh medium. Selection was then initiated with puromycin (Thermo Fisher Scientific). Transfected cells were incubated in medium with 3 μg/mL puromycin for 48 hours. Stably transfected cells were maintained in their regular medium plus puromycin at 1 μg/mL. Knockdown was verified via reverse transcriptase quantitative PCR.

### Real-time quantitative PCR

Total RNA was isolated using an RNeasy Mini Kit (QIAGEN), and cDNA was synthesized with qSCRIPT cDNA Super-mix (Quanta Biosciences). Quantitative PCR reactions were performed using Fast SYBR-Green Mastermix (Thermo Fisher Scientific) on an Applied Biosystems QuantStudio 3 Real-Time PCR system. The threshold cycle (Ct) value for each gene was normalized to the expression levels of *Gapdh*, and relative expression was calculated by normalizing to the ΔCt value of mouse astrocytes, unless otherwise described. The primer sequences were obtained from PrimerBank or previously published papers and were as follows: *GAPDH* forward, 5′-TGGATTTGGACGCATTGGTC-3′, reverse, 5′-TTTGCACTGGTACGTGTTGAT-3′; *ODC1* forward, 5′-TCCTTGATGAAGGCTTTACTGC-3′, reverse, 5′-ACATSAGAACGCATCCTTATCGTC-3′.

### TCGA and GTEx data analysis

Clinical and mRNA expression data for the IDH–wild type subset of the GBM cohort and lower-grade glioma cohorts of The Cancer Genome Atlas (TCGA) were downloaded from the GlioVis portal (http://gliovis.bioinfo.cnio.es); GBM and normal brain cohorts of Genotype-Tissue Expression (GTEx) were downloaded from the GTEx portal (https://gtexportal.org/home/).

### Analysis of single-cell RNA-Seq data from Ruiz-Moreno et al.

The publicly available dataset GBmap was utilized and analyzed using Seurat v4.0 (Ruiz-Moreno et al., ref. 33). The Core GBmap data were downloaded, which comprise 338,564 total cells harmonized from 16 different studies. Briefly, the authors used a semi-supervised neural network model to integrate the data and used any additional data to classify cell type. Furthermore, they used gene modules to further categorize cell types. The Seurat .rds file was downloaded, and the cell type annotations determined by GBmap were used. The average *ODC1* expression per sample was calculated using Seurat’s AverageExpression function. CD8^+^ cytotoxic, CD8^+^ effector memory, and CD8^+^ NK signature cells were aggregated to represent the CD8-expressing cells per tumor. For each sample, the percentage of CD8-expressing cells was calculated, using the total number of cells per sample as the denominator. Spearman’s correlation was calculated and plotted in Figure 7.

### Analysis of Visium spatial transcriptomics data from Ravi et al.

Processed data were downloaded from https://doi.org/10.5061/dryad.h70rxwdmj Deconvolution of spots as described by Ravi et al. (34) was obtained from the authors upon request. We calculated the correlation between the gene expression of interest in each spot and the average proportion of estimated CD8^+^ T cells in all adjacent spots using a simple Pearson’s correlation.

### MALDI-TOF spatial analysis

Flash-frozen tissue was sectioned at a thickness of 10 μm directly onto indium tin oxide–coated glass slides. Frozen sections were dried in a freeze dryer (ModulyoD, Thermo Electron Corp.) for 30 minutes, followed by collection of optical images using the light microscope embedded in a MALDI-TOF mass spectrometry imaging instrument (iMScope QT, Shimadzu) prior to matrix application. α-Cyano-4-hydroxycinnamic acid (CHCA) (C2020) was purchased from MilliporeSigma. Matrix deposition was performed by 2-step deposition method using iMLayer for sublimation and iMLayer AERO (Shimadzu) for matrix spraying. The thickness of the vapor-deposited matrix was 0.7 μm, and the deposition temperature was 250°C. For CHCA matrix spraying, 8 layers of 10 mg/mL CHCA in acetonitrile/water (50:50, vol/vol) with 0.1% trifluoroacetic acid solution were used. The stage was kept at 70 mm/s with 1 second dry time at a 5 cm nozzle distance and pumping pressure kept constant at 0.1 and 0.2 MPa, respectively. MALDI-TOF experiments were performed using an iMScope QT instrument (Shimadzu). The instrument was equipped with a laser-diode-excited Nd:YAG laser and an atmospheric pressure MALDI. Data were collected at 10 μm spatial resolution with positive polarity.

### Bulk RNA-Seq

Normal and tumor regions were dissected from flash-frozen tissue and ground in liquid nitrogen, and RNA was extracted using the RNeasy RNA extraction kit (QIAGEN, 74104). TruSeq libraries (TruSeq RNA Library Prep v2, Illumina) were sequenced on the NextSeq System (Illumina) to produce 132 bp single-end reads.

### GBM patient samples

Frozen GBM specimens were collected by the Cleveland Clinic Rose Ella Burkhardt Brain Tumor and Neuro-Oncology Center after written informed consent was obtained from the patients. The studies were conducted in accordance with recognized ethical guidelines and approved by the Cleveland Clinic Institutional Review Board (IRB 2559). Twenty-three male and a total of 23 patient samples (*n* = 11 female; *n* = 12 male), approximately age-matched, were collected.

### Statistics

GraphPad Prism software (RRID:SCR_002798, version 10, GraphPad Software Inc.) was used for data presentation and statistical analysis. Unpaired or paired, 2-tailed *t* test or 1-way or 2-way ANOVA was used with a multiple-comparison test as indicated in the figure legends. Data represent the mean ± SEM. Where applicable, ROUT outlier test (designed to identify 1 or more outliers in a dataset based on nonlinear regression) was performed on data and identified outliers removed. Survival analysis was performed by log-rank test. *P* values less than 0.05 were considered significant.

### Study approval

All animal procedures were performed in accordance with the guidelines and protocols approved by the Institutional Animal Care and Use Committee at the Cleveland Clinic and by the Walter and Eliza Hall Institute Animal Ethics Committee. Human samples were acquired in accordance with recognized ethical guidelines and approved by the Cleveland Clinic Institutional Review Board (IRB 2559).

### Data availability

Bulk RNA-Seq data were uploaded to the Gene Expression Omnibus database (GEO GSE279139). All other data generated in this study are available in the Supporting Data Values file.

## Author contributions

KEK, JL, DB, and JDL contributed to conception and design. Methodology was developed by KEK, JL, DB, and ZW. Data were acquired by SM, KEK, JL, DB, JB, SD, ERW, SZW, TL, LF, and VN. KEK, JL, DB, ESH, JV, TL, LF, VN, SF, SAB, JRW, and JDL contributed to analysis and interpretation of data. JL, DJS, OR, JSY, SLH, JMB, DB, and JDL contributed to writing and review of the manuscript. SJ, MM, MMG, DJS, and JDL contributed to administrative, technical, or material support. This study was supervised by JDL.

## Supplementary Material

Supplemental data

Supporting data values

## Version 1 11/19/2024

In-Press Preview

## Version 2 01/16/2025

Electronic publication
