# Supplementary material for: Tumor cell–derived spermidine promotes a protumorigenic immune microenvironment in glioblastoma via CD8+ T cell inhibition
Source: J Clin Invest. 2024 Nov 19;135(2):e177824. doi: 10.1172/JCI177824 (PMC11735101; doi:10.1172/JCI177824)
Supplement: Supplemental data [file jci-135-177824-s158.pdf]

Supplementary Figure 1

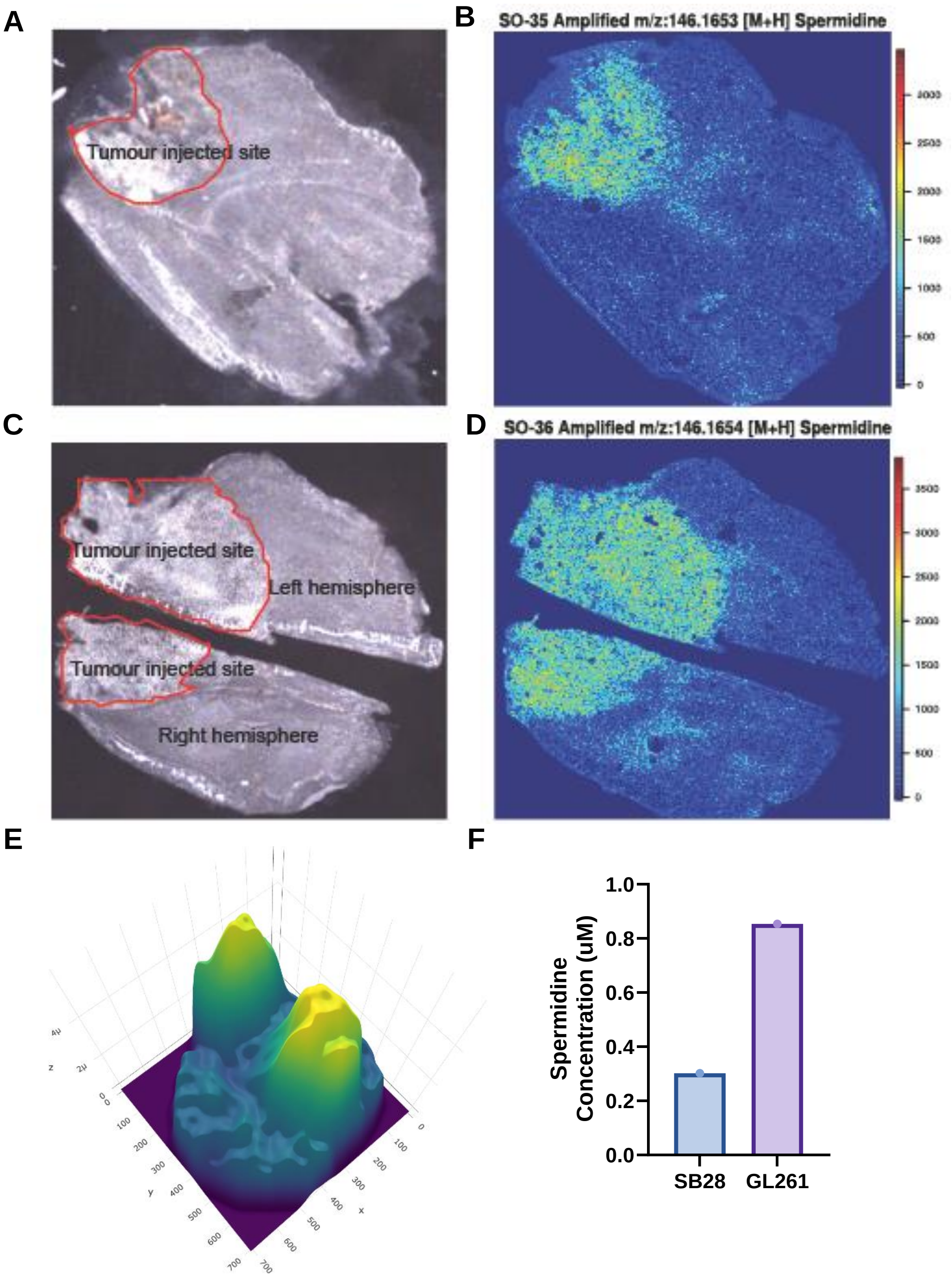

Supplementary Figure 1. Spatial MALDI-TOF analysis of mouse tumors shows high SPD production in the tumor microenvironment. (A-D) After intracranial transplantation with syngeneic GBM cells (GL261), the mouse brain was removed and sectioned for staining and mass spectrometry. (A,C) Identification and delineation of tumor areas in brain tissue. (B,D) SPD levels in the tumor and surrounding brain with corresponding intensity scale. (E) 3D plot of the estimated density of abundance of SPD. (F) Conditioned media secreted SPD measurement via mass spectrometry in syngeneic mouse lines.

Supplementary Figure 2

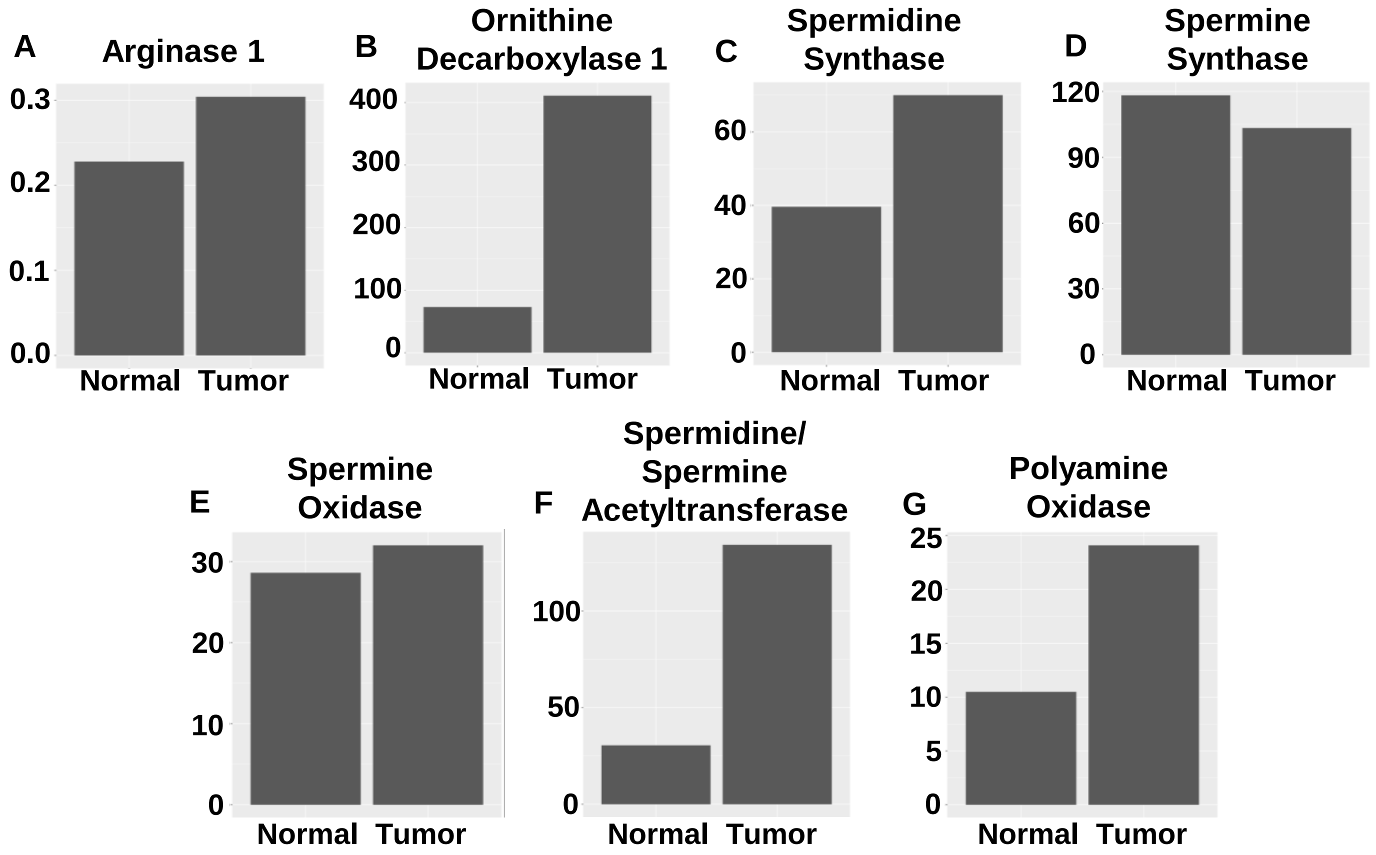

Supplementary Figure 2. Bulk RNAseq data highlights increased polyamine pathway enzymes. (A-E) Tumor tissue and surrounding brain tissue from mice implanted with syngeneic GBM cells (CT2A) were processed via bulk RNA sequencing; expression of genes encoding enzymes of the main *de novo* polyamine synthesis pathway was analyzed.

Supplementary Figure 3

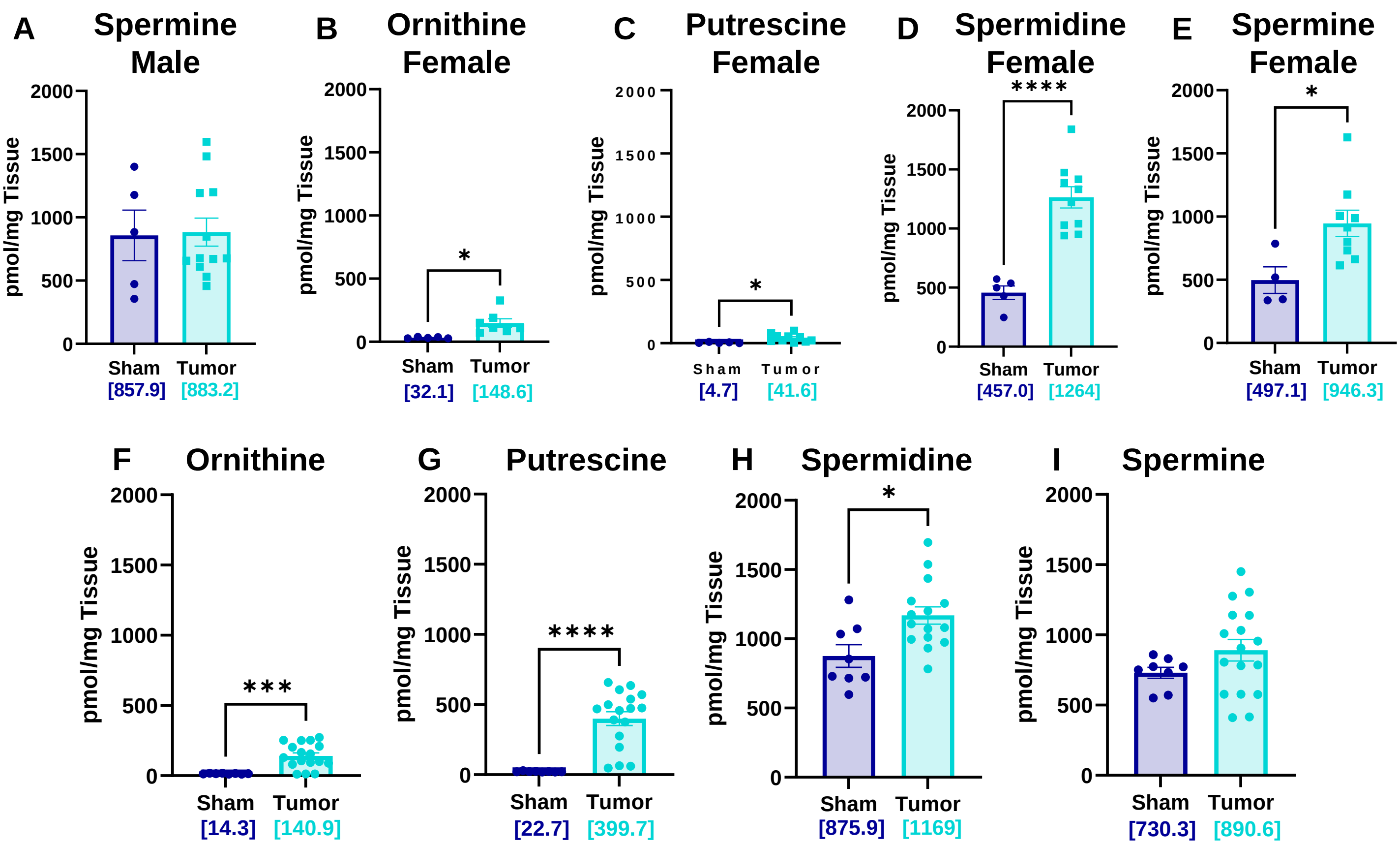

Supplementary Figure 3. SPD levels are increased in females in a mouse GBM model. LC-MS/MS was performed on tumors removed from B6 mice 17 days after intracranial injection of mouse GBM cell line (A) Tumor levels of spermine in males (25K/injection GL261). (B-E) Polyamine measurements in female mice (25K/injection GL261). (F-I) Polyamine measurements in both male and female mice (20K/injection SB28). Statistical significance was determined by unpaired *t*-test (**p*<0.05, ****p<0.001*, *****p*<0.0001). Bracketed numbers indicate mean.

Supplementary Figure 4

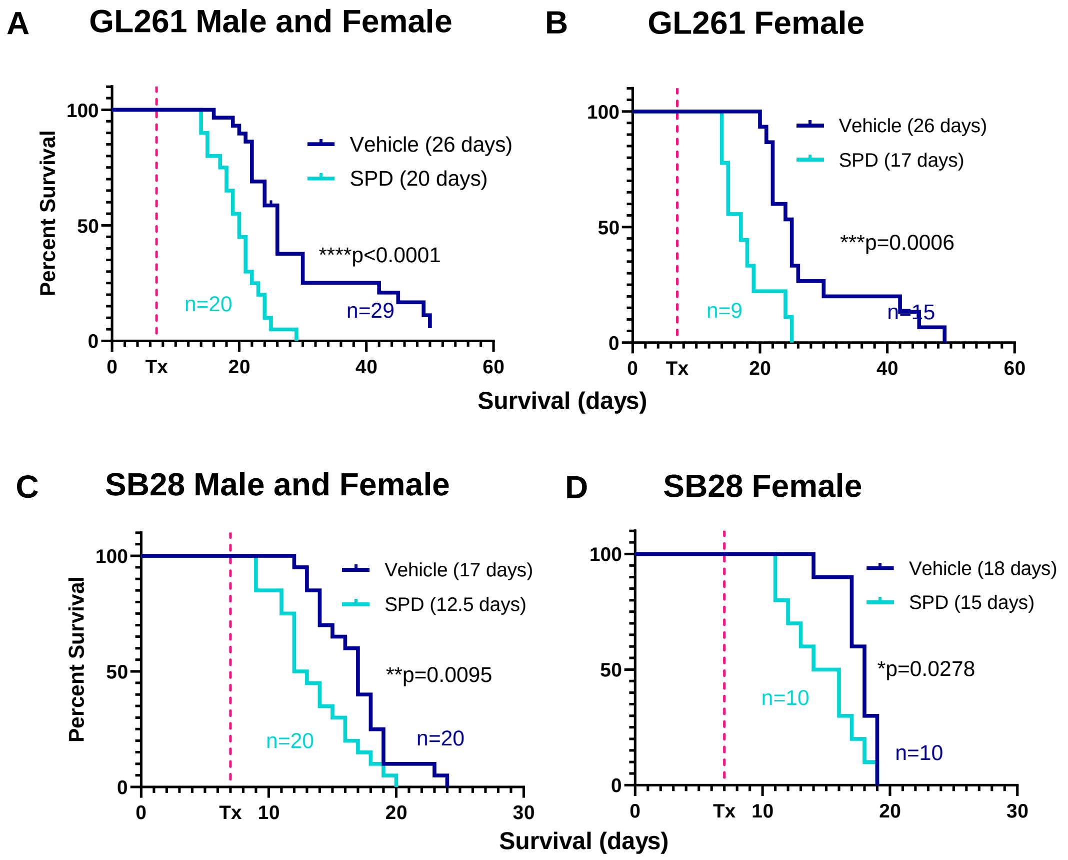

Supplementary Figure 4. Exogenous SPD treatment of both male and female mice reduces survival. (A-D) Survival analysis was performed after intracranial injection of mouse GBM cell lines (25K/injection GL261, 20K/injection SB28) in B6 mice. Median survival days and number of animals are indicated in the figure. Data combined from three independent experiments. Statistical significance was determined by log-rank test, considering *p*-value <0.05 to be significant.

Supplementary Figure 5

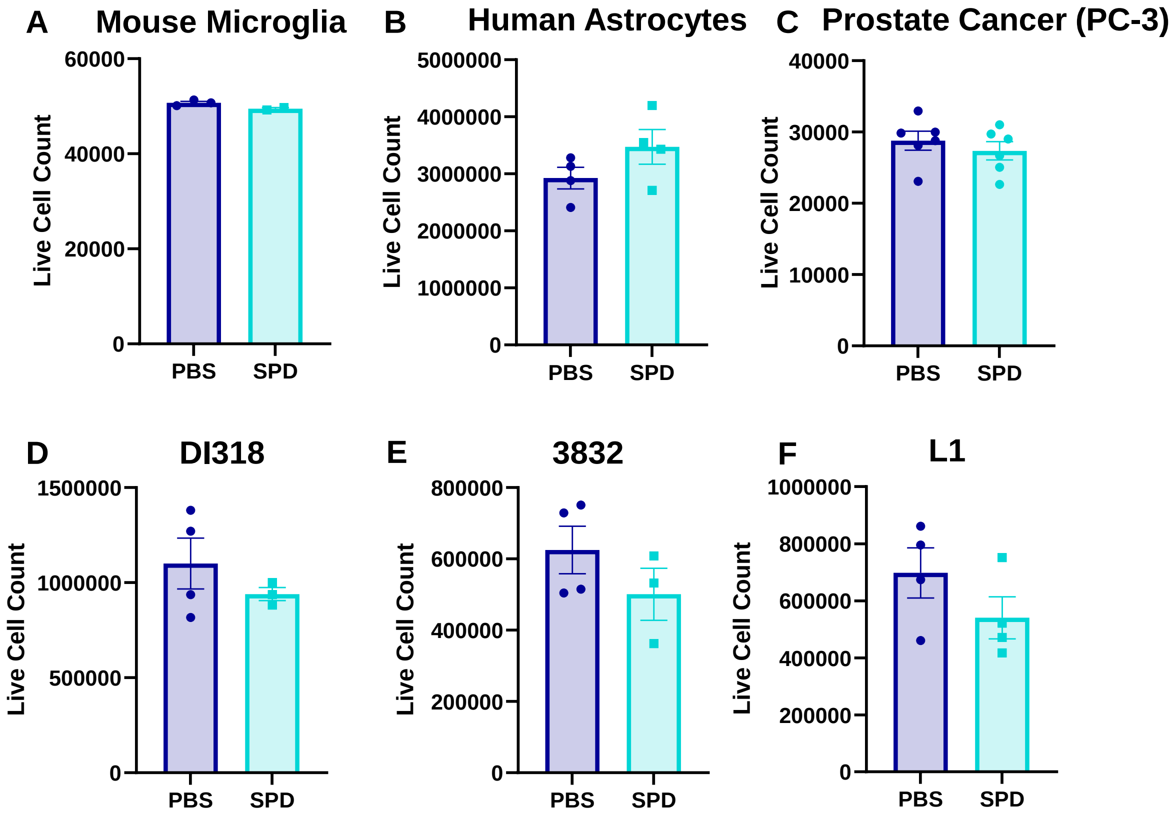

Supplementary Figure 5. SPD does not affect viability of resident brain cells or human tumor cells *in vitro*. (A-F) Cells treated with 5uM SPD or vehicle *in vitro*. (A-B) Resident brain cells. (C) Human prostate cancer cells. (D-F) Human-derived GBM models.

Supplementary Figure 6

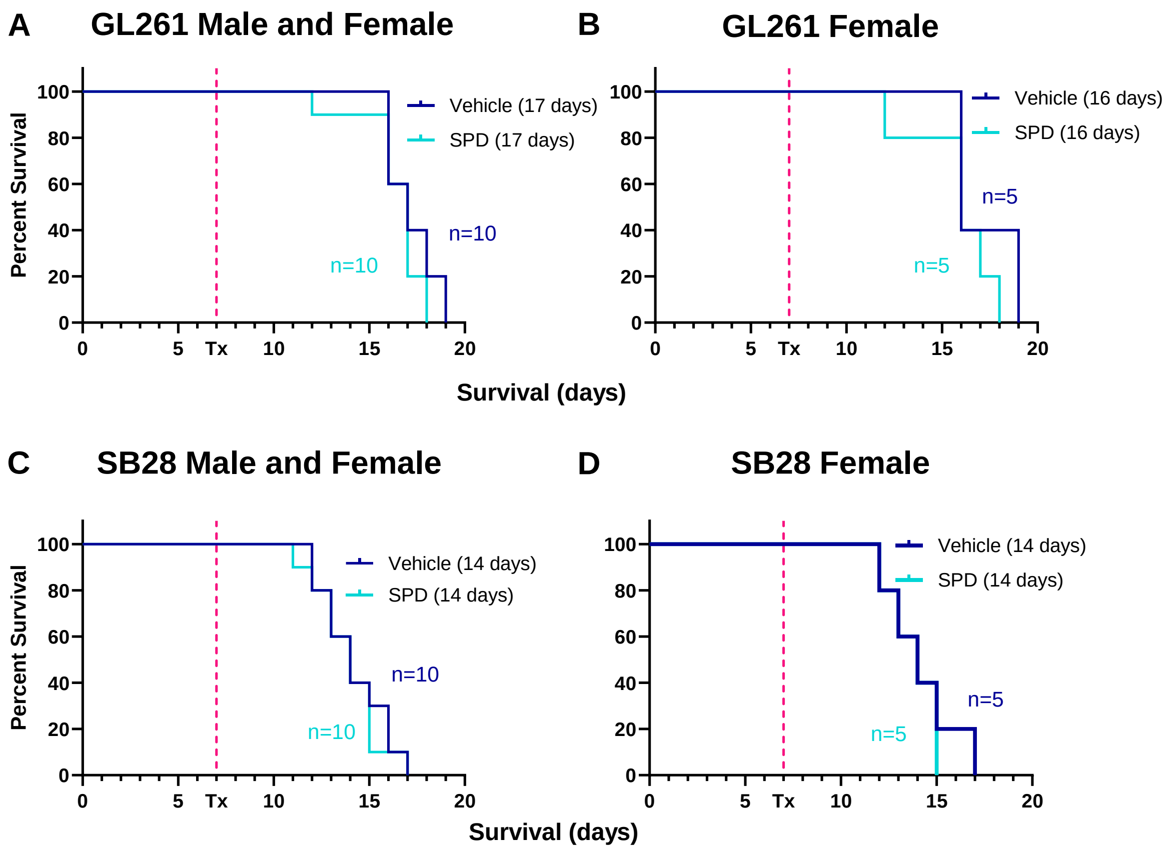

Supplementary Figure 6. SPD interacts with the immune system to drive GBM progression in both male and female mice. (A-D) Survival analysis was performed after intracranial injection of mouse GBM cell lines (25K/injection GL261, 20K/injection SB28) in immunocompromised NSG mice. Median survival days and number of animals are indicated in the figure. Statistical significance was determined by log-rank test, considering *p*-value <0.05 to be significant.

Supplementary Figure 7

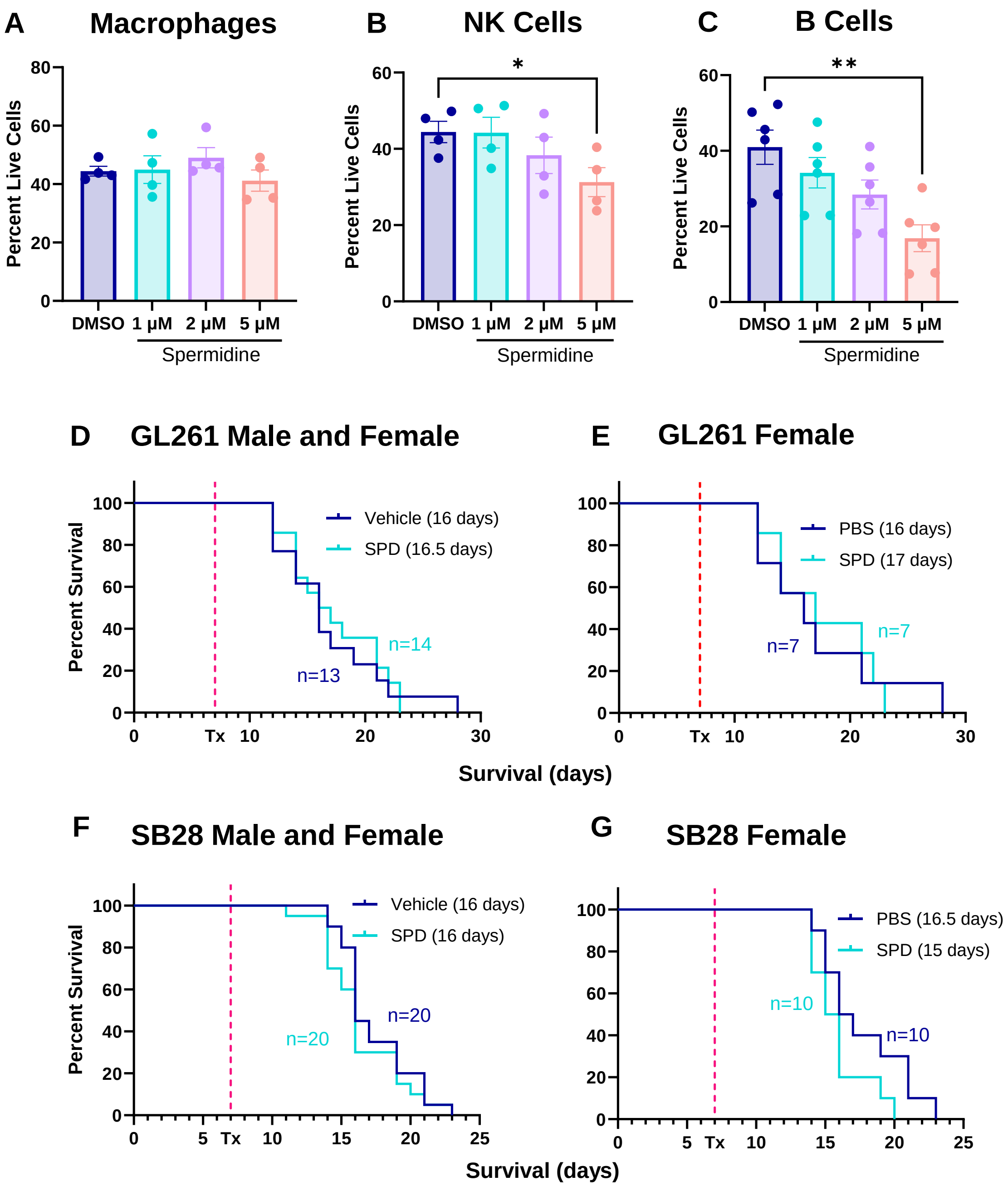

Supplementary Figure 7. Lymphocyte subsets are affected by SPD. (A-C) Splenocyte-derived lymphocyte subsets were treated with physiological levels of SPD *in vitro*. (D-G) Survival analysis was performed after intracranial injection of mouse GBM cell lines (25K/injection GL261, 20K/injection SB28) followed by 50 mg/kg IP SPD treatment or PBS vehicle in Rag1 knockout mice. Median survival days and number of animals are indicated in the graph. Data combined from two independent experiments. Statistical significance for (A-C) was determined by one-way ANOVA (**p*<0.05, ***p*<0.01). Statistical significance for (D-G) was determined by log-rank test, considering *p*-value <0.05 to be significant.

Supplementary Figure 8

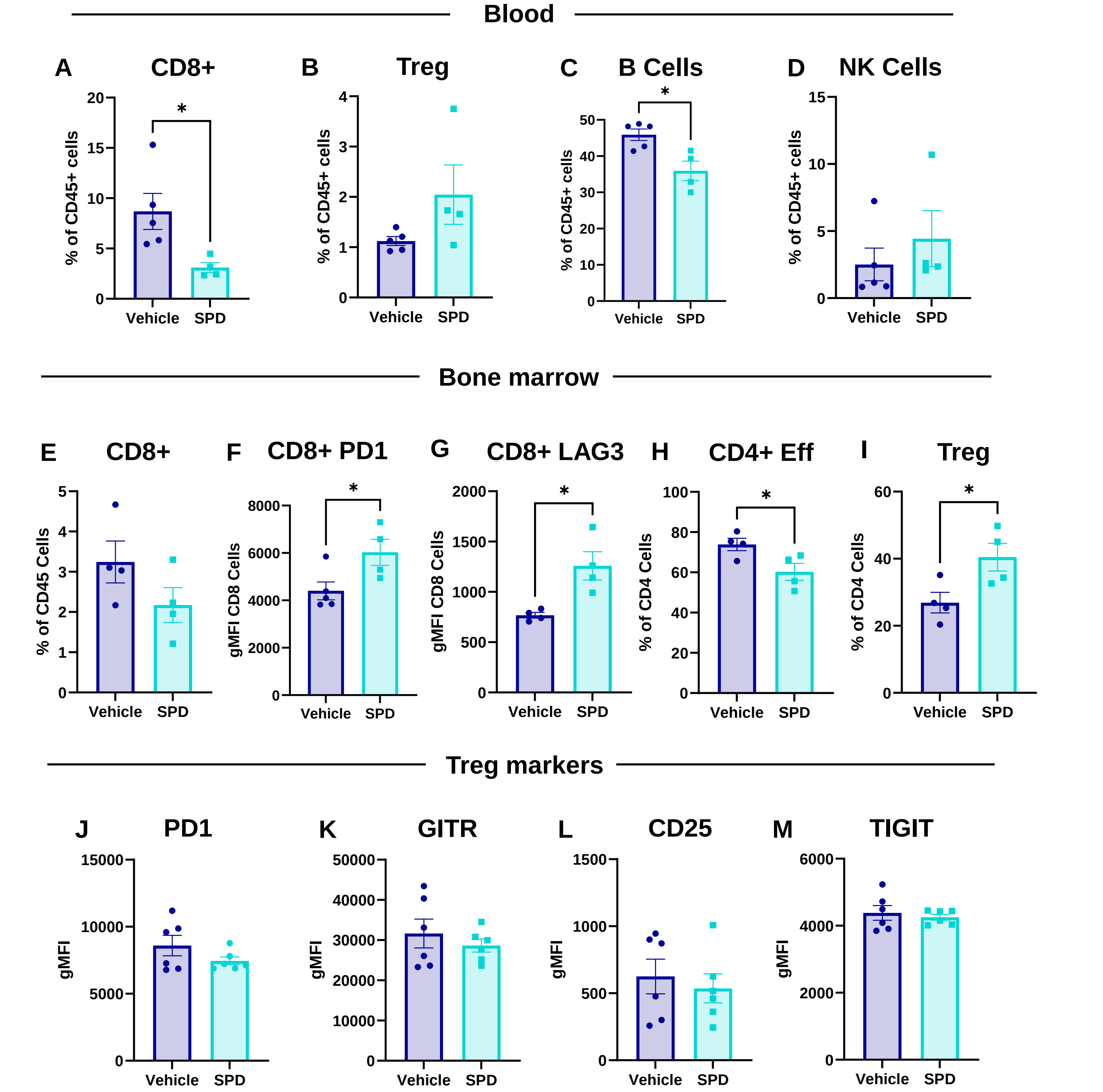

Supplementary Figure 8. Effects of SPD on other immune compartments. After intracranial injection of mouse GBM cell line SB28 (20K/injection) into male B6 mice followed by 50 mg/kg SPD IP treatment or PBS vehicle, the tumor-bearing hemisphere was collected and processed for flow cytometry immune phenotyping. (A-D) Frequency of various immune cell types in blood. (E) Proportion of CD8+ T cells in bone marrow. (F-G) Exhaustion markers of CD8+ T cells. (H-I) Proportion of effector cells and Tregs in CD4+ population. (J-M) Treg markers. Statistical significance for (A-M) was determined by unpaired *t*-test (*$p<0.05$).

Supplementary Figure 9

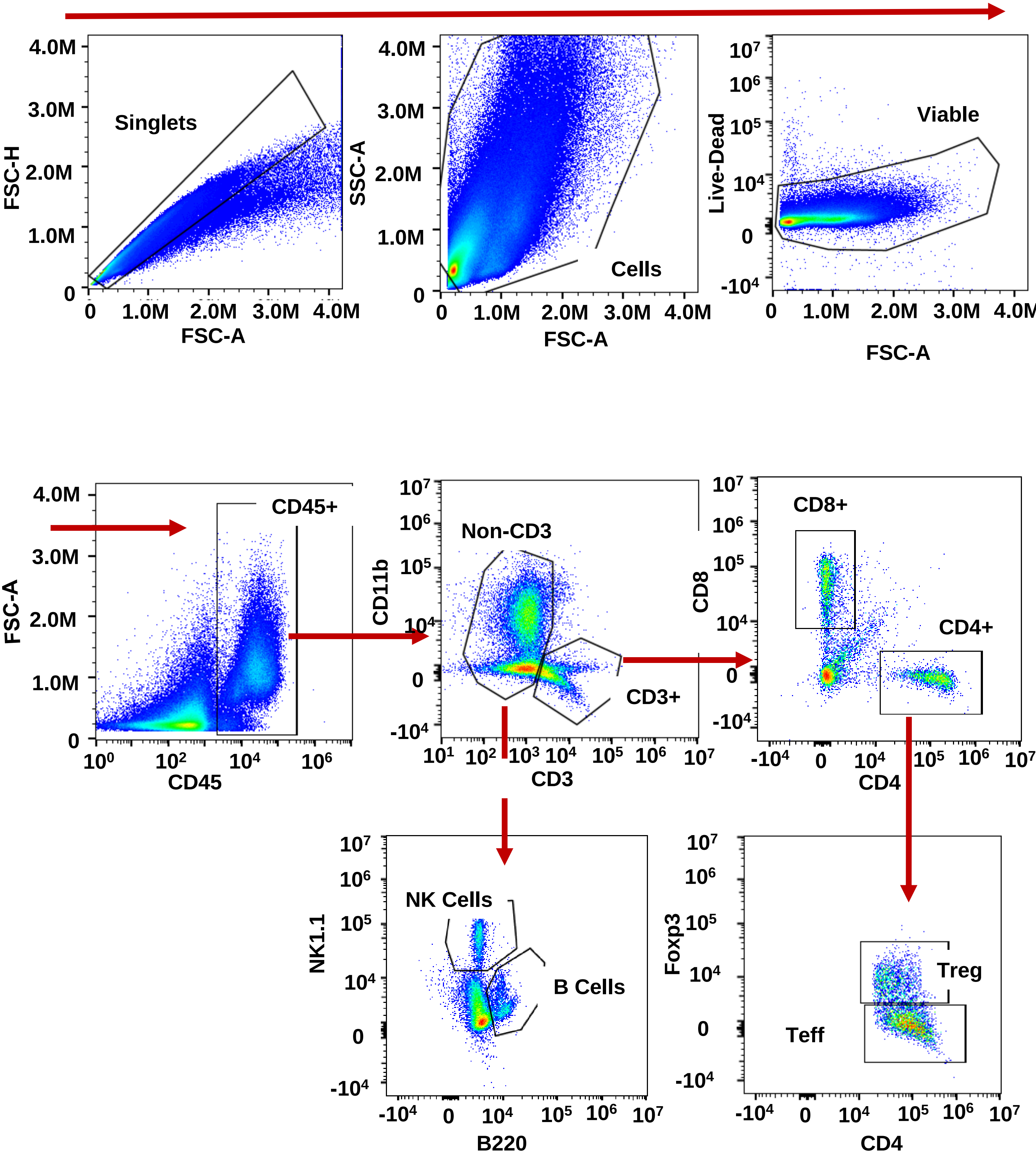

Supplementary Figure 9. Representation of flow cytometry gating.

Supplementary Figure 10

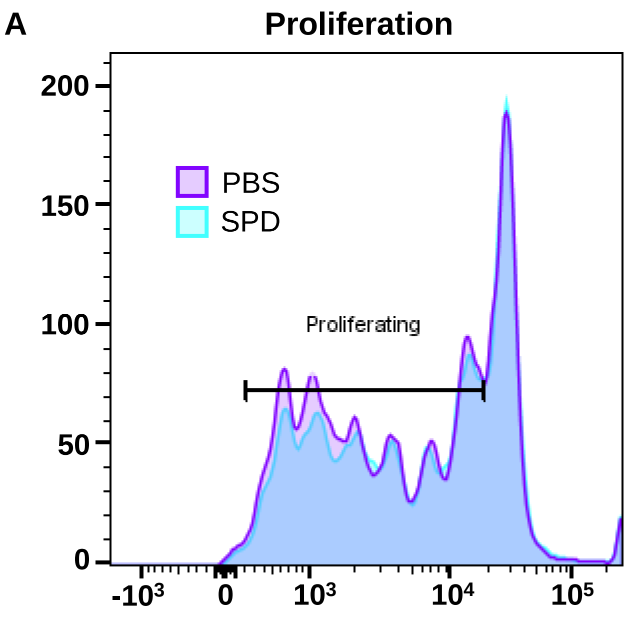

Supplementary Figure 10. CD8+ T cells do not exhibit proliferation changes in the presence of SPD. Splenocyte-derived CD8+ T cells were treated with SPD or PBS vehicle *in vitro*. (A) CD8+ cells stained with CellTrace Violet and analyzed with flow cytometry; percentage of proliferating cells indicated by gate.

Supplementary Figure 11

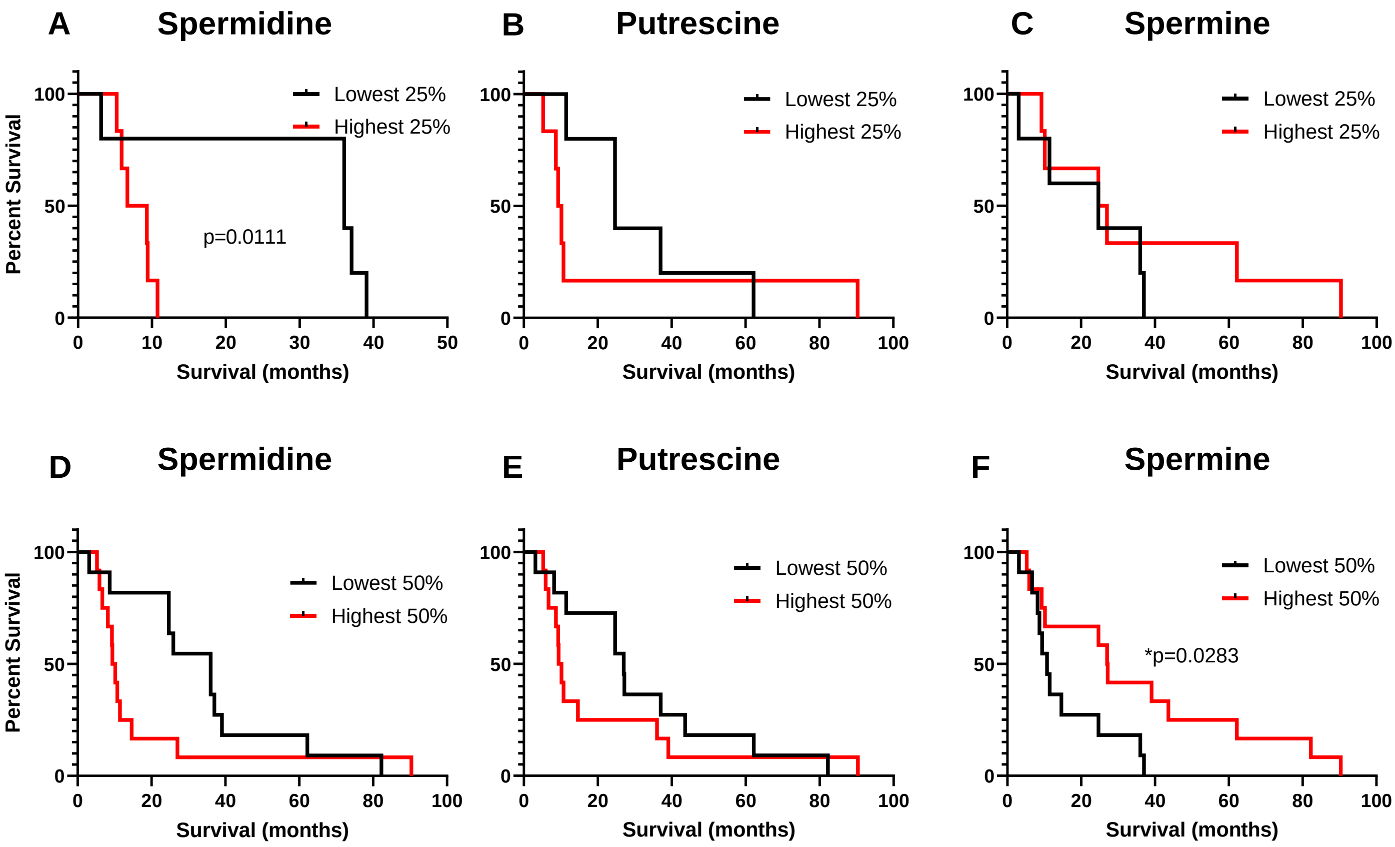

Supplementary Figure 11. Members of polyamine pathway measured in GBM patient tumors in relation to overall survival. (A-C) Tumor tissue from primary resection of GBM patients, survival of highest quartile of polyamine levels compared to lowest quartile; metabolites measured via LC-MS/MS. (D-F) Tumor tissue from primary resection of GBM patients, survival of above median and below median polyamine measurements; metabolites measured via LC-MS/MS. Statistical significance was determined by log-rank test.
